# A review of the *Cercyon* Leach (Coleoptera, Hydrophilidae, Sphaeridiinae) of the Greater Antilles

**DOI:** 10.3897/zookeys.681.12522

**Published:** 2017-06-21

**Authors:** Emmanuel Arriaga-Varela, Matthias Seidel, Albert Deler-Hernández, Viktor Senderov, Martin Fikáček

**Affiliations:** 1 Department of Zoology, Faculty of Science, Charles University, Prague, Viničná 7, CZ-128 44 Praha 2, Czech Republic; 2 Department of Entomology, National Museum, Cirkusová 1, CZ-193 00 Praha, Czech Republic; 3 Pensoft Publishers, Prof. Georgi Zlatarski Street 12, 1700 Sofia, Bulgaria; 4 Institute of Biodiversity and Ecosystems Research, Bulgarian Academy of Sciences, 1113 Sofia, Bulgaria

**Keywords:** Megasternini, morphology, taxonomy, new species, Caribbean, COI, DNA barcode, larva, biodiversity informatics

## Abstract

The representatives of the genus *Cercyon* Leach occurring in the Greater Antilles are reviewed. Ten species are recorded, of which five are described here as new: *C. gimmeli* **sp. n.** (Dominican Republic), *C. armatipenis* **sp. n.** (Dominican Republic), *C. taino* **sp. n.** (Dominican Republic), *C. sklodowskae* **sp. n.** (Jamaica) and *C. spiniventris* **sp. n.** (Dominican Republic). Diagnoses and detailed distributional data are also provided for *C. floridanus* Horn, 1890 (distributed in southeastern United States of America and Cayman Islands), *C. insularis* Chevrolat, 1863 (endemic to the Antilles) *C. praetextatus* (Say, 1825) (widely distributed in the New World incl. Greater Antilles), *C. quisquilius* (Linnaeus, 1761) (an adventive species of Paleartic origin) and *C. nigriceps* (Marshall, 1802) (an adventive species probably of Oriental origin). *Cercyon armatipenis, C. gimmeli, C. taino* form a group of closely related species only distinguishable by male genitalia and DNA sequences. A key to the Great Antillean *Cercyon* is provided and important diagnostic characters are illustrated. The larvae of *C. insularis* and *C. taino* were associated with adults using COI barcode sequences, illustrated and diagnosed. Full occurrence data, additional images and COI barcode sequences were submitted to open access on-line depositories in an effort to provide access to complete data.

## Introduction

Until very recently, water scavenger beetles (Hydrophilidae) from the Greater Antilles in the Caribbean Region were largely neglected and systematic or faunistic studies were scarce. For instance, 46 of 56 hydrophilid species recorded from Cuba were described in the 18^th^ and 19^th^ centuries (Peck 2005). Nevertheless, recent taxonomic studies have brought to light many new species and new country records in this region e.g. in genera *Berosus* Leach (Deler-Hernández et al. 2013a), *Enochrus* Thomson (Deler-Hernández and Delgado 2010; Short 2005), *Oosternum* Sharp (Deler-Hernández et al. 2014), *Phaenonotum* Sharp (Deler-Hernández et al. 2013b) and *Tropisternus* Solier (Spangler and Short 2008). However, many other groups still await a comprehensive treatment, which includes *Cercyon*, the most speciose genus within the subfamily Sphaeridiinae. As with most members of the subfamily, *Cercyon* species have predominantly terrestrial habits, and are frequently associated with decaying plant material and feces. Approximately 260 species have been described from all zoogeographical zones (Short and Fikáček 2011), of which 24 have been recorded from Central and South America (Hansen 1999; Fikáček 2006). The number of described species seems highly underestimated in the Neotropical Region, due to the lack of recent taxonomic work. The most comprehensive identification resource for Central American fauna still remains the iconic *Biologia Centrali-Americana* (Sharp 1882, 1887). The situation is also aggravated by the presence of introduced Old World synanthropic species, which are difficult to recognize and are sometimes confused with native species (Fikáček 2009).

Six species of *Cercyon* have been recorded from the Greater Antilles: the Cuban-endemic *C. insularis* Chevrolat whose identity has remained unclear (Hansen 1999; Peck 2005), two species widely distributed in the New World (*Cercyon variegatus* Sharp, *Cercyon praetextatus* (Say); Leng and Mutchler 1917; Smetana 1984; Fikáček 2009), one species native to southeastern United States of America (*Cercyon floridanus* Horn; Thomas et al. 2013) and two widely distributed adventive species (*Cercyon nigriceps* Marsham, *Cercyon quisquilius* (Linnaeus); Leng and Mutchler 1917; Fikáček 2009). Our recent field work and examination of museum material revealed that additional species occur in the area, which provided the impetus for this study. In order to provide a review that will constitute a reliable reference for future studies on the genus *Cercyon* and the tribe Megasternini, we complemented the traditional taxonomic account with COI sequences (i.e., “DNA barcodes”), complete occurrence data, and full set of high-resolution photographs, all deposited in online freely available platforms.

## Material and methods

**Examined specimens and depositories.** A total of 848 specimens of *Cercyon* from the Greater Antilles were examined, including the type specimens of *Cercyon insularis* Chevrolat and *C. variegatus* Sharp. Label data are only reproduced verbatim for type specimens; each individual label is separated by double slash “//”; notes on the label data or additional information are written between square brackets []. All holotypes are marked with red label bearing the following text: “HOLOTYPE, *Cercyon* [name of the species] sp. n., Arriaga-Varela, Seidel, Deler-Hernández and Fikáček des. 2016“. All paratype specimens are marked by yellow label bearing the following text: “PARATYPE, *Cercyon* [name of the species] sp. n., Arriaga-Varela, Seidel, Deler-Hernández and Fikáček des. 2016”. A georeferenced dataset of the studied specimens is available as Excel spreadsheet in Suppl. material 1. The file only includes specimens identified to species. The distribution maps (Figs 15–16) were constructed from the GPS data extracted from the Excel spreadsheet and mapped using the R script (see Suppl. material 4).

The examined specimens are deposited in the following collections:

**BCPC** Bruno Clarkson private collection, Rio de Janeiro, Brazil;

**BMNH** Natural History Museum, London, United Kingdom (M.V.L. Barclay);

**CMN** Canadian Museum of Nature, Ottawa, Canada (R. Anderson, F. Génier);

**CNC** Canadian National Collection of Insects, Arachnids and Nematodes, Ottawa, Canada (P. Bouchard);

**CNIN** Colección Nacional de Insectos, Instituto de Biología, Universidad Nacional Autónoma México, Mexico City, Mexico (S. Zaragoza);

**FSCA** Florida State Collection of Arthropods, Gainesville, USA (P. Skelley);

**HNHM** Hungarian National History Museum, Budapest, Hungary (O. Merkl, G. Szél);

**MNHNSD** Museo Nacional de Historia Natural, Santo Domingo, Dominican Republic (C. Suriel);

**MNHN** Muséum National d’Histoire Naturelle, Paris, France (A. Mantilleri);

**NHMW** Naturhistorisches Museum, Wien, Austria (M. A. Jäch);

**NMPC** National Museum, Prague, Czech Republic (M. Fikáček);

**SBNM** Santa Barbara Museum of Natural History, Santa Barbara, USA (M. L. Gimmel);

**SBPC** Stewart Peck Personal Collection, Ottawa, Canada;

**UPRM** University of Puerto Rico, Mayagüez, Puerto Rico (A. Segarra);

**ZMUC** Zoological Museum, Natural History Museum of Denmark, Copenhagen, Denmark (A. Solodovnikov).

**Morphological studies.** Specimens were dissected, with genitalia embedded in a drop of alcohol-soluble Euparal resin on a piece of glass glued to a small piece of cardboard attached below the respective specimen. All species are diagnosed and illustrated, and new species are described in detail.

Habitus photographs were taken using a Canon D-550 digital camera with attached Canon MP-E65mm f/2.8 1–5 macro lens. Pictures of genitalia were taken using a Canon D1100 digital camera attached to an Olympus BX41 compound microscope; pictures of different focus were combined in Helicon Focus software. Scanning electron micrographs were taken using Hitachi S-3700N environmental electron microscope at the Department of Paleontology, National Museum in Prague. Pictures used for plates were adapted in Adobe Photoshop CS6. All original pictures including additional views not presented in this paper are published and freely available on Flickr in order to serve for further morphological studies.

**DNA barcoding.** Most of the examined specimens were collected during recent expeditions to Cuba, Dominican Republic and Puerto Rico. Samples were preserved in 96% ethanol and stored at -20 °C. DNA was extracted from complete specimens using a QiaGen Blood and Tissue DNA extraction kit following the manufacturer's instructions. The highly variable 5’ region of the mitochondrial cytochrome c oxidase subunit I gene (COI) was amplified using LCO1490 (5'-GGTCAACAAATCATAAAGATATTGG-3') and HCO2198 (5'-TAAACTTCAGGGTGACCAAAAAATCA-3') primers (Folmer et al. 1994). Each 10 µl PCR reaction contained 6.7 µl H_2_O, 0.4 µl of MgCl_2_ (25 mM), 0.2 µl of dNTPs (10 mM), 0.3 µl of each forward and reverse primer (10 µM), 0.1 µl of Taq polymerase (5 u/µl), 1.0 µl of 10x Taq buffer, and 1.0 µl of DNA template. The PCR conditions consisted of 3 min at 94 °C + 35 cycles of 30 s at 94 °C, 45 s at 48 °C and 1 min at 72 °C + 8 min at 72 °C. 5 µl of each PCR product were purified by adding 0.5µl (20 u) Exonuclease I (Exo1) and 1µl (1 u) Thermosensitive Alkaline Phosphatase (FastAP) (Thermo Fisher Scientific) and incubating the mixture for 15 min at 37°C, followed by 15 min at 80°C. The Sanger sequencing was performed by BIOCEV (Vestec, Czech Republic) on a capillary DNA sequencer. Sequences were edited with Geneious 9.1.4. We did not attempt DNA extraction and sequencing of old museum specimens, which is why we only provide sequences for six of the ten species occurring in Greater Antilles.

**Analyses of molecular data.** In order to identify the larvae collected along with adult specimens, a maximum likelihood analysis of obtained COI sequences was performed. We combined the newly generated sequences of freshly collected adults and larvae, and combined them with additional sequences of two introduced species (*C. quisquilius* and *C. nigriceps* from Europe and Canada) from the Barcode of Life Data Systems (BOLD; http://www.boldsystems.org). Sequences were aligned using the ClustalW algorythm in Geneious 9.1.4. The final alignment had a length of 610 bp and was tested for the best nucleotide substitution model using MEGA7 (Kumar et al. 2016). A phylogenetic analysis using the maximum likelihood algorythm and 1000 bootstrap replicates was performed in the same software.

### Open access to complete data

This taxonomic paper includes only a part of the data accumulated in the course of our study. Part of the primary data (e.g., unedited photographs, the complete set of unedited SEM micrographs, DNA sequences, spreadsheet-fomatted species distribution data) are not included here. To make all primary data accessible, we deposited them to open access on-line depositories as specified below. For more details about biodiversity data publishing, see e.g. the policies and guidelines implemented for Pensoft Publishers (Penev et al. 2011).

***Complete primary data*.** Complete primary data were submitted as a .zip file to the Zenodo depository (https://zenodo.org/) under the doi 10.5281/zenodo.580260.

***Species distribution data*.** The distribution data on all specimens examined are presented in unstructured text format directly in the paper. The conversion of these data into a structured, computable format (as XML, so called parsing) is difficult, and no algorithm exists for parsing occurrence records (see Sautter and Böhm 2014 for the analogous problem of parsing literature references).

The text-formatted distribution data published here are, however, based on a structured Excel spreadsheet following the Darwin Core (henceforth DwC) format for biodiversity data described by Wieczorek et al (2012). DwC defines how the data should be structured (i.e. which columns may be included in the table, how they should be called and which part of the data they should include). In some cases DwC also specifies the format of the entries (e.g., how date should be formatted). Details are available through the website of the Biodiversity Information Standards (TWDG; http://rs.tdwg.org/dwc/terms/). Being a formal biodiversity standard, DwC is nowadays used by a wide spectrum of on-line biodiversity portals, e.g. by the Global Biodiversity Information Facility (GBIF), Encyclopedia of Life, and the Atlas of Living Australia. This is the reason why we selected it. The Excel file is attached here as Suppl. material 1, it is included in the .zip file submitted to Zenodo, and was used for the GBIF submission.

The Publication of distribution data to GBIF is possible through the institution or organization, which is a member or partner of GBIF (direct submissions from individual users are not possible) using the Integrated Publishing Toolkit (IPT). IPT allows to upload the distribution data from the DwC-formatted Excel spreadsheet, specify the metadata about the dataset, and publish the data to the GBIF portal. We submitted our data through Pensoft as an organization associated with GBIF using the Pensoft IPT Data Hosting Centre (http://ipt.pensoft.net/).

***DNA data and voucher information.*** The cytochrome oxidase I barcode sequences and the data about the voucher specimens were submitted to the Barcode of Life Data Systems (BOLD; http://www.boldsystems.org/) using the user web interface available after registration. The submission requires first the submission of the specimen data using the Excel-based spreadsheet following the Specimen Data Submission Protocol (http://www.boldsystems.org/index.php/resources/handbook?chapter=3_submissions.html&section=data_submissions). To prevent the re-typing of the specimen data again, we wrote an R script converting the data from DwC to the format required for BOLD submissions (see Suppl. material 3). Once specimen data are submitted, all other information (voucher photos, DNA sequences, DNA trace files) can be submitted, using the identification code (SampleID) to connect the data to the respective voucher specimen. We submitted the sequences under Process ID GANTC001-16 and GANTC002-17 to GANTC015-17 in BOLD.

***Original photo-documentation.*** The original photo-documentation includes the unedited high-resolution versions of photos and SEM micrographs that we used in this publication, plus many photos and SEM micrographs that were taken for comparative purposes but are not published here. We submitted all these files to Zenodo as a part of the .zip file containing all our primary data. Since the images are not easy to see in this way, we also submitted all photos to Flickr photo hosting service where they can be easily displayed; they are available at https://www.flickr.com/photos/142655814@N07/collections/72157678126129411/.

## Taxonomy

### Cercyon

Taxon classificationAnimaliaColeopteraHydrophilidae

Leach, 1817

Cercyon Leach, 1817: 95. - Type species: Dermestes melanocephalus Linnaeus (designated by Thomson 1859: 19).

#### Diagnosis.

*Cercyon* can be distinguished from other hydrophilid genera occurring in the Greater Antilles by the following combination of characters: antenna with compact club; prothorax with conspicuous antennal groove not reaching pronotal margin; medial part of prosternum not demarcated from lateral parts; metaventrite without arcuate lines in anterolateral corners; mesoventral plate fusiform, narrowing anteriorly and posteriorly, touching anterior margin of metaventrite in one point.

*Cercyon* species are very similar to the members of *Pelosoma*, a Neotropical genus that is recorded from the Lesser Antilles; *Pelosoma* differs from *Cercyon* by the mesoventral plate widely contacting the metaventrite (it only narrowly contacts it in *Cercyon*). Small species of *Cercyon* may resemble the members of *Oosternum*, which can be easily distinguished from *Cercyon* by possessing a metaventrite with an arcuate ridge delimiting its anterolateral corner, and in some species also by elevated median part of the prosternum.

#### Key to the Greater Antilles species of *Cercyon*

**Table:** 

1	Small species, body length 1.0–2.1 mm. Metaventrite with complete femoral lines (Fig. 13d)	***Cercyon nigriceps* (Marsham**)
–	Larger species, body length 2.3–4.1 mm. Metaventrite without femoral lines (Figs 7d, 8g, 9c, h, 10g, 11g, 12g, 13b, f)	**2**
2	Mesoventral plate very wide, 1.9× as long as wide (Fig. 7d). Mesoventral plate and pentagonal raised part of the metaventrite with large, deep, semicircular punctures (Fig. 7d)	***Cercyon floridanus* Horn**
–	Mesoventral plate moderately to very narrow, 3.3–5.9× as long as wide (Figs 8f, 9c, h, 10f, 11f, 12f, 13b, e). Mesoventral plate and pentagonal raised part of the metaventrite with small and shallow punctures (Figs 8g, 9c, h, 10g, 11g, 12g, 13b, f)	**3**
3	Mesoventral plate wide, 3.3× as long as wide (Fig. 13a). Dorsal coloration black, with large yellow spot at elytral apex; lateral margin of elytra narrowly yellow, the yellow stripe not widened in humeral area (Fig. 2g–i)	***Cercyon praetextatus* (Say)**
–	Mesoventral plate narrow, 5.7–6.3× as long as wide as long as wide (Figs 8f, 9c, h, 10g, 11g, 12g, 13e). Dorsal coloration variable, if elytra is black with a large yellowish spot at apex, then the yellow coloration at the lateral margin of elytra expands to humeral area	**4**
4	Metaventrite with raised pentagonal area markedly wide at midlength, 0.6× as long as wide (Fig. 13f)	***Cercyon quisquilius* (Linnaeus)**
–	Metaventrite with raised pentagonal area rather narrow at midlength, 0.9–1.2× as long as wide (Figs 8g, 9c, h, 10g, 11g)	**5**
5	Dorsal surface of head black, with reddish-brown spot(s) at vertex. Pronotum (Fig. 4a) yellowish to dark reddish, with a large median spot and smaller spot on each side, often fused together into one large tri-lobate spot. Elytra brown with black humeral spots extending to anterior margin and suture. Prosternum with median ridge not projected ventrally at anterior margin (Fig. 12c)	***Cercyon insularis* Chevrolat**
–	Dorsal surface of head including vertex black, anterolateral margins of clypeus yellowish. Pronotum either black with yellowish to reddish lateral margins (Figs 1a–i, g, 2a–c), or uniformly light brown (Fig. 3a–i). Elytra either more or less uniformly brown, or black with yellowish to reddish lateral and apical parts	**6**
6	Pronotum uniformly light brown, elytra more or less uniformly greyish-brown (Fig. 3a). First abdominal ventrite of females (Fig. 11h) with longitudinal carina prolonged into an acute spiniform setose process; longitudinal carina of male first abdominal ventrite not projected. Raised area of metaventrite comparatively wide, 0.8× as long as wide (Fig. 11g)	***Cercyon spiniventris* sp. n.**
–	Pronotum and elytra black with pale contrasting markings (Figs 1a, c, d, f, g, i, 2a, c): pronotum black with creamy-white or reddish lateral margins; elytra black with creamy-white to reddish humeral spot, lateral margins and apical third. First abdominal ventrite of both sexes not projecting beyond posterior margin (Figs 8h, 10h). Raised area of metaventrite rather narrow, 1.1–1.2× longer than wide (Figs 8g, 9c, h)	**7**
7	Prosternum with median ridge forming a small rounded to weakly pointed process (Fig. 10c). Raised median area of metaventrite reaching anterior margin of metaventrite (Fig. 10g). Anterior margin of mentum emarginate medially (Fig. 10a). Apex of fifth abdominal ventrite with a triangular bulged projection in females (Fig. 10i), not modified in males. Jamaica	***Cercyon sklodowskae* sp. n.**
–	Prosternum with median ridge forming a large rounded knob (Figs 8c, 9g). Raised median area of metaventrite not reaching anterior margin of metaventrite (Figs 8g, 9c, h). Anterior margin of mentum not emarginate medially (Figs 8a, 9a, h). Apex of fifth abdominal ventrite rounded in both sexes. Hispaniola	***Cercyon gimmeli* species group**...**8**
8	Median lobe strongly acuminate at apex (Fig. 5f–g); parameres distinctly shorter than phallobase (Fig. 5e)	***Cercyon taino* sp. n.**
–	Median lobe blunt at apex (Fig. 5c, k); parameres as long as or longer than phallobase (Fig. 5a, i)	**9**
9	Median lobe wide basally, narrowing apically, with large gonopore and many spines in apical fifth (Fig. 5j–k); parameres ca. as long as phallobase (Fig. 5i)	***C. armatipenis* sp. n.**
–	Median lobe narrowly parallel–sided, with indistinct gonopore and without spines at apex (Fig. 5b–c); parameres distinctly longer than phallobase (Fig. 5a)	***C. gimmeli* sp. n.**

### Species treatments

#### *Cercyon gimmeli* species group

This species group is composed of three very closely related species endemic to the island of Hispaniola. Specimens are indistinguishable on the basis of external morphology and can be only told apart by examination of male genitalia. At least in one locality, two species of this group were collected syntopically. For this reason, we refrain from using female specimens as paratypes unless the specimen was associated with males by DNA barcode, and female specimens are not listed in the text below neither in the DarwinCore spreadsheet submitted to GBIF for that reason. Below, we provide a diagnosis allowing to separate all species of the species group from other *Cercyon* species. Further on, we describe *Cercyon gimmeli* sp. n. in full, and provide only comparative diagnoses for the other two species, *C. armatipenis* sp. n. and *C. taino* sp. n.

**Figure 1.**
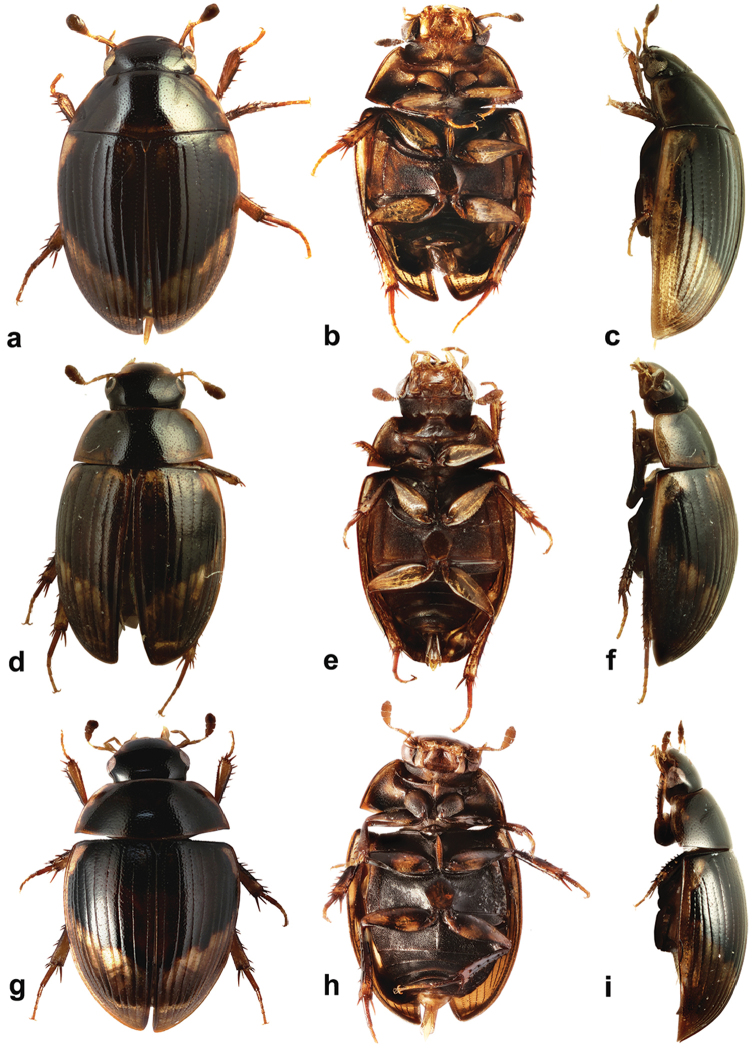
*Cercyon* spp. n. **a–c** *Cercyon gimmeli* sp. n. **d–f** *Cercyon armatipenis* sp. n. **g–i** *Cercyon taino* sp. n. **a, d, g** dorsal habitus **b, e, h** ventral habitus **c, f, i** lateral habitus.

**Diagnosis of the *Cercyon gimmeli* species group.** Members of the species group can be differentiated from other Greater-Antillean *Cercyon* by the following combination of characters: size 2.8–3.5 mm; dorsal surface of head black, with yellowish anterolateral margins of clypeus; pronotum black with sharply defined creamy-white areas at lateral margins; elytra black, with large, pale, rather sharply defined spot in posterior third of both elytra (Fig. 1); anterior margin of mentum not emarginated (Figs 8a, 9a, f); medial ridge of prosternum anteriorly forming a rounded knob (Figs 8c, 9g); mesoventral plate narrow, ca. 5.7× as long as wide (Figs 8f, 9c, h); metaventrite (Figs 8g, 9c, h) without femoral lines, with narrow raised pentagonal area, 1.1× as long as wide; first abdominal ventrite without spine-like process in both sexes (Fig. 8h); metatibia slightly bent outwards; apex of fifth abdominal ventrite without an apical triangularly bulged projection in both sexes (Fig. 8i).

By the dorsal coloration, the species of *C. gimmeli* group could be confused with *C. praetextatus* (Say), *C. floridanus* Horn and *C. sklodowskae* sp. n. However, they can be easily distinguished from from *C. praetextatus* and *C. floridanus* Horn by the distinctly narrower mesoventral plate (compare Fig. 8f with Figs 7d and 13a) and yellow coloration of lateral part of elytra expanding to humeral area at elytral base, and from *C. sklodowskae* sp. n. by the bare median portion of mesoventrite not reaching anteriorly, bulged projection of the prosternum, metatibia slightly bent outwards, anterior margin of mentum not emarginated, and females without a triangularly bulging projection at apex of the fifth ventrite.

**Distribution.** The species group is endemic to Hispaniola and seems widespread on the island. No records are known from Haiti, likely due to collecting bias.

##### Cercyon gimmeli sp. n.

Taxon classificationAnimaliaColeopteraHydrophilidae

http://zoobank.org/DDB83C0C-AAF4-4BE7-BD49-E86443ADD650

Figures 1a–c , 5a–d , 8a–i , 15a

###### DNA barcodes.

GANTC002-17 to GANTC006-17

###### BIN ID.

BOLD:ADF7790.

###### Figures on Flickr.

www.flickr.com/photos/142655814@N07/albums/72157671425298360

###### Type locality.

Dominican Republic, Samaná Province, Monumento Natural Salto, El Limón 2.8 km SSW of El Limón, 19°16.56'S 69°26.47'W, 160 m a.s.l.

###### Type material.

**Holotype** (male): “DOMINICAN REP.: Samaná, MN Salto El Limón 2.8 km SSW of El Limón; 19°16.56'S 69°26.47'W; 160 m; 2.ix.2014, Deler, Fikáček, Gimmel. DR29a // secondary vegetation and tiny remnants of forest among coffee plantations and pastures: in horse excrement” (NMPC) [DNA extract: MF1256.1]. **Paratypes: DOMINICAN REPUBLIC: Samaná**: same label data as the holotype (1 males, 1 female: NMPC; 1 male: BCPC; 1 male: CNC; 1 male: CNIN; 1 male: SBNM) [DNA extract: MF1256.2 in NMPC]. **La Vega**: “DOMINICAN REP.: La Vega, PN A. Bermúdez, 8 km W of Manabao, 19°4.05'N, 70°51.98'W, 1140 m, 22–26.viii.2014, Deler, Fikáček, Gimmel DR16 // montane broad-leaf forest: in cow and horse excrement” (2 males: NMPC). “DOMINICAN REPUBLIC: Pr. La Vega La Cienaga de Manabao, Park Hdqt, 3-5-VII-1999, 3000 ft, R.E. Woodruff, blacklight” (1 male: FSCA). **Monseñor Nouel**: “DOMINICAN REP.: Msñ. Nouel, PN La Humeadora; 11.6km SSW, of Piedra Blanca; 18°44.92'N, 70°21.63'W; 636 m; 8.ix.2014, Deler, Fikáček, Gimmel DR41 // in horse excrement in moist broad-leaf forest in a valley of a small stony stream” (14 males, 2 females: NMPC; 1 male: BMNH; 1 male: CMN; 2 males: MNHNSD; 1 male: NHMW; 3 males: SBNM) [DNA extracts of both females: MF1217.1, MF1217.2 in NMPC]. **Barahona**: “DOMINICAN REP.: Prov. Barahona. nr. Filipinas, Larimar Mine: 26-VI/7-VII-1992: Woodruff & Skelley, flight trap” (2 males: FSCA; 2 males: NMPC). “DOMINICAN REP.: Prov. Barahona. nr. Filipinas, Larimar Mine: 26-VI/7-VII-1992: Woodruff, Skelley, Skillman. dung trap” (10 males: FSCA; 4 males: NMPC). “DOMINICAN REP.: Prov. Barahona. nr. Filipinas, Larimar Mine: 20-VII/26-VI-1992: Woodruff & Skelley, human dung” (7 males: FSCA). “DOMINICAN REP.: Prov. Barahona. nr. Filipinas, Larimar Mine: 26-VI/7-VII-1992: Woodruff, Skelley & Skillman, at light” (5 males: FSCA). “DOMINICAN REP.: Prov. Barahona. nr. Filipinas, Larimar Mine: 20-VII/26-VI-1992: Woodruff & Skelley, at day” (2 males: FSCA). DOMINICAN REP.: Prov. Barahona. nr. Filipinas, Larimar Mine: 20-VII/26-VI-1992: Woodruff & Skelley, at light” (5 males: FSCA). “DOMINICAN REP.: Prov. Barahona. nr. Filipinas, Larimar Mine: 26-VI/7-VII-1992: Woodruff & Skelley, rat carrion” (1 male: FSCA). “DOMINICAN REP.: Prov. Barahona. nr. Filipinas, Larimar Mine: 26-VI/7-VII-1992: Skelley, day catch, beating” (1 male: FSCA).

###### Diagnosis.

Externally identical with other members of the *Cercyon gimmeli* species group, it may be only distinguished from them by the morphology of the aedeagus (Fig. 5a–d): parameres longer than the phallobase; median lobe without spines, narrowly parallel-sided, with rounded apex.

###### Description.

*Body.* (Fig. 1a–c) 2.8–3.5 mm long (length of holotype: 3.25 mm); long oval, 1.8–1.9× as long as wide, widest at basal fourth of elytra; moderately convex, 3.2× as long as high, (height of holotype: 1.05 mm). *Coloration*. Dorsal surface of head black, clypeus with widely yellowish anterolateral margins. Antennae and ventral surface of head, including mouthparts, brown, antennal club dark-brown. Pronotum black, with a wide, rather sharply defined creamy-white area along lateral margins, broader at anterior half. Prosternum and hypomeron brown, gradually turning black in posterior half. Elytra dark brown to black, with large, pale, rather sharply defined apical spot covering posterior quarter of elytral interval 1, and gradually larger portion on subsequent intervals up to posterior three–quarters of interval 9; two lateralmost intervals completely pale; apical area slightly darker (yellowish brown) posteriorly, with bright yellowish stripe or spots at least along its anterior border; basal portion of elytra with a pair of pale elongated dots at sides of scutellar shield. Ventral surface of mesothorax blackish to pitchy black. Metepisternum brown. Metaventrite brown with dusk anteromedial part. Abdomen brown, ventrites sometimes with dusky marks on anterior margin. Legs yellow to brown ventrally, dorsally with black markings on femora.

*Head.* Clypeus with rather sparse and shallow punctation consisting of crescent-shaped setiferous punctures intermixed with denser, smaller and rather transverse non–setiferous punctures; interstices without microsculpture. Anterior margin of clypeus with a narrow bead. Frontoclypeal suture conspicuous as a zone without punctuation, vanished in middle. Frons with punctation similar to that on clypeus, punctures of same shape all over, slightly sparser on sides; interstices without microsculpture. Eyes rather small; interocular distance about 6× the width of one eye in dorsal view. Labrum membranous, nearly completely concealed under clypeus, only with narrowly exposed sinuate anterior margin. Mentum (Fig. 8a) subtrapezoid, widest at posterior fourth, about 2.1× wider than long, 1.3× wider at widest part than at anterior margin, concave in anterior half, with a shallow transverse impression anteromesally; surface glabrous, punctures rather small and shallow, almost vanishing anteromessally, interstices without microsculpture. Antenna with 9 antennomeres, scapus ca. 1.7× as long antennomeres 2–6 combined; antennal club moderately elongate, about twice as long as wide, about as 1.1× as long as scapus; antennomere 9 acuminate at apex.

*Prothorax.* Pronotum transverse, widest at base 2.1–2.2× wider than long; 1.8× wider at base than between anterior angles, 1.8× wider than head including eyes, as convex as elytra in lateral view. Punctation moderately dense and shallow, consisting of crescent-shaped setiferous punctures intermixed with denser, smaller and rather transverse non–setiferous punctures; punctures slightly feebler on sides. Prosternum (Fig. 8b) strongly tectiform medially, medial ridge thickened in anterior half, forming a large rounded knob in lateral view (Fig. 8c). Antennal grooves distinct, with lateral margin curved, slightly feebler anteriorly.

*Pterothorax.* Scutellar shield 1.1× as long as wide, sparsely punctured. Elytra widest at anterior fifth, 2.55–2.85× as long as pronotum, 1.25–1.35× as wide as pronotum; surface glabrous (Fig. 8d), with 10 series of punctures; series 6, 8 and 9 not reaching elytral base, serial punctures getting slightly larger laterally; intervals moderately convex; punctation of interval 1 and odd intervals composed of crescent-shaped setiferous punctures intermixed with denser, smaller and rather transverse non–setiferous punctures; even intervals with non-setiferous punctures only; all interstices without microsculpture. Humeral bulge indistinct. Mesoventral plate (Fig. 8f) narrowly elongate, ca. 5.7× as long as wide, widest at midlength, gradually and symmetrically narrowing posteriad and anteriad to pointed apices, posterior tip slightly overlapping over anterior portion of metaventrite; surface with few sparsely arranged coarse punctures. Metaventrite with narrow median raised pentagonal area (Fig. 8g), 1.1× as long as wide at widest portion, glabrous, weakly and sparsely punctuate, punctures with fine setae at least along margins of elevation, with bare area not reaching anterior margin; femoral lines absent; lateral parts of metaventrite densely covered by short pubescence.

*Legs.* Femora with sparse rather shallow punctures ventrally, interstices with weak microsculpture at bases, consisting of longitudinal lines; tibial grooves distinct. Tibiae with rather large lateral spines. Metatibiae moderately narrow and elongate, slightly bent outwards, 0.3–0.4× as long as elytra, 5.3× as long as wide. Metatarsus long, 0.9× as long as metatiba, with few short but rather stout setae ventrally.

*Abdomen* with five ventrites, first abdominal ventrite about as long as second and third ventrites combined, with distinct median longitudinal carina (Fig. 8h) narrowing posteriad, not projecting posteriorly in both sexes; fifth ventrite (Fig. 8i) with acuminate and very weakly bulged apex in both sexes.

*Male genitalia.* Median projection of sternite 9 (Fig. 5d) very narrow, shorter than lateral struts, without subapical setae. Aedeagus: Phallobase (Fig. 5a) distinctly shorter than parameres, asymmetrically narrowing basally, base widely rounded. Parameres narrow throughout, slightly widened apically, subsinuate on outer face near apex, apex pointed, with a couple of setae. Median lobe (Fig. 5b) narrow throughout, indistinctly narrowing apically; apex (Fig. 5c) rounded with finely truncate tip, gonopore moderately large, situated subapically; basal portion with dorsal plate narrow and simply bifid basally. Median projection of sternite 9 (Fig. 5a) very narrow, shorter than lateral struts, without subapical setae.

###### Variability.

In some specimens the pale spots at sides of the scutellar shield are longer, almost reaching the second fourth of elytral length.

###### Etymology.

We are pleased to dedicate this species to Matthew L. Gimmel (Santa Barbara Museum of Natural History), who participated in the expedition to the Dominican Republic and collected part of the type series of this species.

###### Distribution.

Dominican Republic: Barahona, La Vega, Monseñor Nouel, Samaná (Fig. 14a).

###### Biology.

All specimens were collected in in broad-leaf tropical forests and coffee plantations and pastures, mainly on cow and horse dung, but also on human dung, rat carrion, at black-light or at day by beating.

##### Cercyon armatipenis sp. n.

Taxon classificationAnimaliaColeopteraHydrophilidae

http://zoobank.org/B937F9C3-3948-4256-8879-37EF7A6ACCBC

Figures 1d–f , 5i–l , 9a–d , 15a

###### DNA barcodes.

GANTC013-17, GANTC014-17

###### BIN ID.

BOLD:ADF5573

###### Figures on Flickr.

www.flickr.com/photos/142655814@N07/albums/72157676345486243

###### Type locality.

Dominican Republic, Independencia Province, Parque Nacional Sierra de Neiba, 11.3 km NW of La Descubierta, 18°39.81'N, 71°46.17'W, 1650 m a.s.l.

###### Type material.

**Holotype** (male): “DOMINICAN R.: Independencia, PN Sierra de Neiba, 11.3 km NW of La Descubierta; 1650 m, 18°39.81'N, 71°46.17'W; 18.viii.2014, Deler, Gimmel DR13 // disturbed montane cloud forest with many ferns and mosses: in cow excrement” (NMPC). **Paratypes: DOMINICAN REPUBLIC: Independencia**: same label data as the holotype (2 males: NMPC; 1 male: BMNH; 1 male: SBNM) [DNA extractions MF1264.5, MF1264.6 in NMPC].

###### Diagnosis.

Externally identical with other members of the *Cercyon gimmeli* species group, it may be only distinguished from them by the morphology of the aedeagus (Fig. 5i–l): parameres as long as phallobase; median lobe moderately wide basally, narrowing apicad, with spines in apical fifth, apex finely truncate.

###### Description.

*Measurements.* (Fig. 1d–f) 3.0–3.7 mm long (length of holotype: 3.45 mm); 1.8–1.9× as long as wide, 3.2× as long as high (height of holotype: 1.15 mm).

Conforming to the description of *C. gimmeli*, with the following differences: *Pterothorax.* Punctation of even intervals consisting of small non-setiferous punctures, but here and there with infrequent crescent-shaped larger setiferous punctures; only lateralmost elytral interval completely pale.

*Male genitalia.* Median projection of sternite 9 (Fig. 5l) moderately wide, slightly widening apically, with few subapical setae. Phallobase (Fig. 5i) as long as parameres, asymmetrically narrowing basally, base narrowly rounded. Parameres wide basally, gradually narrowing towards apex, sinuate on outer face near apex, apex rounded, with 3 apical and few subapical setae. Median lobe (Fig. 5j) wide basally, gradually narrowing towards apex; (Fig. 5k) apex finely truncate, with numerous backward-directed spines on dorsal and lateral surfaces; gonopore large, subapical; basal portion with dorsal plate simply bifid.

###### Etymology.

The species name is derived from Latin words *armatus* (armed) and *penis* (penis), in reference to the diagnostic character of this species, i.e. the apex of the median lobe armed by small spines.

###### Distribution.

Dominican Republic: Independencia (Fig. 15a).

###### Biology.

Specimens were collected in cow dung in a cloud forest.

##### Cercyon taino sp. n.

Taxon classificationAnimaliaColeopteraHydrophilidae

http://zoobank.org/7EEF52BE-5EAE-412C-AE23-DD1AB8CA8C1A

Figures 1g–i , 5e–h , 9f–i , 15b

###### DNA barcodes.

GANTC007-17 to GANTC009-17

###### BIN ID.

BOLD:ADF5574

###### Figures in Flickr.

www.flickr.com/photos/142655814@N07/albums/72157671656199632

###### Type locality.

Dominican Republic, Samaná Province, dam 2.5 km N of Samaná, 58 m a.s.l., 19°13.70'N, 69°19.85'W.

###### Type material.

**Holotype** (male): “DOMINICAN REP.: Samaná, dam 2.5 km N of Samaná, 19°13.70'N, 69°19.85'W; 58 m, 5.ix.2014, Deler, Fikáček, Gimmel DR35 // in older cow excrements dampered by recent rains at the grassy bank of a reservoir” (NMPC) [DNA extract: MF1735]. **Paratypes: DOMINICAN REPUBLIC: La Vega**: “DOMINICAN REP.: La Vega, PN Valle Nuevo, Salto Aguas Blancas; 18°50.60'N, 70°40.68'W; 1655 m; 25.viii.2014, Deler, Fikáček, Gimmel DR21 // Sifting of moist leaf litter in small remnants of montane forest in a small ravine with a spring and on slopes just above the small river” (5 males: NMPC); “DOMINICAN REP.: La Vega, PN A. Bermúdez, 10.3 km W of Manabao, 19°4.37'N, 70°53.26'W, 1270 m, 26.viii.2014, M. Fikáček lgt. (DR22) (2 males: NMPC). **Independencia**: “DOMINICAN R.: Independencia, PN Sierra de Neiba, 11.3 km NW of La Descubierta; 1650 m, 18°39.81'N, 71°46.17'W; 18.viii.2014, Deler, Gimmel DR13 // disturbed montane cloud forest with many ferns and mosses: in cow excrement” (1 male: NMPC; 1 male: BMNH; 1 male: SBNM) [DNA extract: MF 1264.1 in NMPC].

###### Diagnosis.

Externally identical with other members of the *Cercyon gimmeli* species group, it may be only distinguished from them by the morphology of the aedeagus (Fig. 5e–g): parameres shorter than phallobase; median lobe without spines, narrowly parallel–sided, pointed at apex.

###### Description.

*Measurements.* (Fig. 1g–i) 2.8–3.5 mm long (length of holotype: 3.35 mm); 1.8–1.9× as long as wide, 3.2× as long as high (height of holotype: 1.10 mm).

Conforming with description of *C. gimmeli*, with the following differences: *Pterothorax*. Punctation of even intervals consisting of small non-setiferous punctures, but here and there with infrequent crescent-shaped larger setiferous punctures; only lateralmost elytral interval completely pale.

*Male genitalia.* Median projection of sternite 9 (Fig. 5h) moderately wide, slightly widening apically, with few subapical setae. Phallobase (Fig. 5e) longer than parameres, asymmetrically narrowing basally, base widely rounded. Parameres narrow throughout, slightly narrowing towards apex, weakly sinuate on outer face near apex, apex rounded, with one apical and one subapical seta. Median lobe (Fig. 5f) narrowly parallel-sided throughout, apically narrowing into pointed apex (Fig. 5g), apex without spines, gonopore minute, subapical; basal portion with dorsal plate simply bifid.

###### Etymology.

The new species is named after the indigenous Taíno people inhabiting the Greater Antilles including Hispaniola before and at the time of the arrival of Europeans.

###### Distribution.

Dominican Republic (Samaná, La Vega, Independencia) (Fig. 15b).

###### Biology.

Examined specimens and the associated larvae (see below) were collected from leaf litter of montane forests and from cow dung.

##### Cercyon sklodowskae sp. n.

Taxon classificationAnimaliaColeopteraHydrophilidae

http://zoobank.org/6679B59D-E7CE-4D95-8894-12C6084607A4

**Figure 2.**
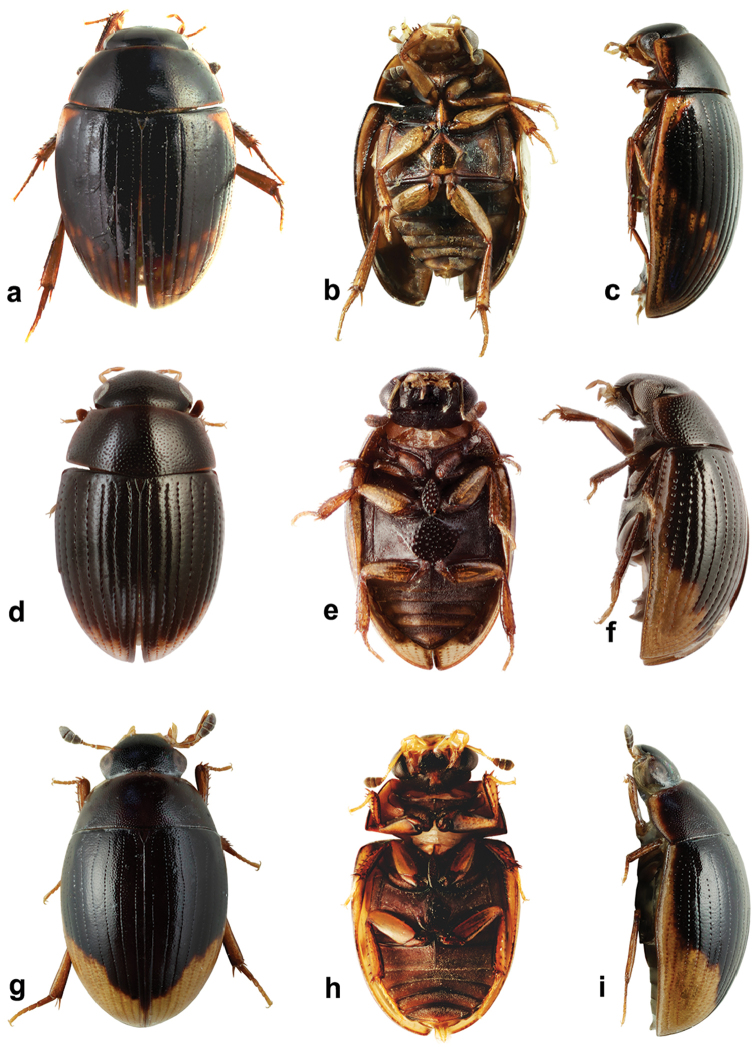
*Cercyon* spp. **a–c** *Cercyon sklodowskae* sp. n. **d–f** *Cercyon floridanus* Horn **g–i** *Cercyon praetextatus* Say **a, d, g** dorsal habitus **b, e, h** ventral habitus **c, f, i** lateral habitus.

Figures 2a–c , 5m–p , 10a–10 , 1 , 15c

###### Figures in Flickr.

www.flickr.com/photos/142655814@N07/albums/72157669507211493

###### Type locality.

Jamaica, Saint Thomas Parish, Corn Puss Gap, 6.44 km N of Bath. 640 m a.s.l.

###### Type material.

**Holotype** (male): “JAM., St. Thomas, Corn Puu Gap [sic!, = Corn Puss Gap], 2100’, 4mi. N, Bath, 3-8.VIII.1974, S. Peck, dung” (CNC). **Paratypes: JAMAICA: St. Thomas**: same data as the holotype (1 female: CNC; 1 female: NMPC); “JAM., St. Thomas, Corn Puss Gap, 2100”, 4mi. N, Bath, 3.viii.74, S. Peck, DT16-20” (1 male: CNC); “JAM., St. Thomas P. Portland Gap, 5500‘,17.XII.72-1.I.73, S&J Peck, cloud for., dung&carrion tr.” (1 male, 1 female: CNC); “JAM., St. Thomas P., Portland Gap, 17.XII.72-1.II.73, S & J Peck (1 female: CNC); “JAM., St. Thomas, below Port. Gap, 1-5.VIII.1974, 4500”, S. Peck, dung trap 12” (1 female: CNC).

###### Diagnosis.

*Cercyon sklodowskae* sp. n. can be easily differentiated from other Greater-Antilles *Cercyon* species by the following combination of characters: body size 3.1–3.5 mm; dorsal surface of head black with yellowish anterolateral margins of clypeus; pronotum black with sharply defined yellowish areas at lateral margins (Fig. 2c); elytra (Fig. 2a) black, with large apical spot covering posterior third; medial ridge of prosternum anteriorly forming a small rounded to weakly pointed projection (Fig. 10c); mesoventral plate (Fig. 10f) narrow, ca. 5.8× as long as wide; metaventrite (Fig. 10g) without femoral lines, with narrow raised pentagonal area, 1.2× as long as wide; first abdominal ventrite without spiniform process in both sexes (Fig. 10h); apex of fifth abdominal ventrite with a triangular projection in females (Fig. 10i); aedeagus with very short parameres (Fig. 5m), median lobe (Fig. 5o) covered with spines in apical third.

*C. sklodowskae* may be confused with *C. praetextatus* (Say) and *Cercyon floridanus* Horn and the members of *C. gimmeli* species group. It can be distinguished from *C. praetextatus* and *C. floridanus* by the head with widely yellowish clypeal margin and yellow coloration of elytra expanded basally (clypeus black and basal part of elytra only narrowly yellow in *C. praetextatus* and *C. floridanus*) and by much narrower mesoventral plate (5.8× as long as wide in *C. sklodowskae*, 1.9× as long as wide in *C. floridanus* and 3.3× in *C. praetextatus*). It differs from members of *C. gimmeli* group by the mesoventral plate reaching anterior part of metaventrite (not reaching anterior margin in *C. gimmeli* group), only a small rounded process of median prosternal ridge (large rounded knob in *C. gimmeli* group), straight metatibia (slightly curved outside in *C. gimmeli* group), and last abdominal ventrite in females forming a triangular projection (without projection in *C. gimmeli* group). It differs from all these species by the morphology of male genitalia; by the apically spinose median lobe it resembles *C. armatipenis*, but differs from it by parameres much shorter than phallobase.

###### Description.

*Body.* (Fig. 2a–c) 3.10–3.45 mm long (length of holotype: 3.29 mm); long oval, 1.8–1.9× as long as wide, widest at basal fourth of elytra; moderately convex, 2.8–3.0× as long as high, (height of holotype: 1.1 mm). *Coloration*. Dorsal surface of head blackish to pitchy black, clypeus with wide rather sharply defined yellowish area along anterolateral margins, slightly broader at sides. Ventral surface of head dark brown, almost black on sides. Antennae, mentum and mouthparts dark brown. Pronotum blackish to pitchy black, with narrowly brownish lateral margins, broader at anterolateral corners (Fig. 2c). Prosternum and hypomeron black, with darker anterolateral margins. Elytron (Fig. 2a) black, with large, pale, rather sharply defined apical spot covering posterior quarter of elytral interval 1 and gradually larger portion on subsequent intervals up to posterior three–quarters on interval 9, lateralmost interval completely yellowish to dark brown; apical spot slightly darker (yellowish-brown) posteriorly, with lighter brown spots at least along its anterior border. Ventral surface of mesothorax (Fig. 2b) blackish to pitchy black. Metepisternum dark brown. Metaventrite black with darker anteromedial part and anterior margins. Abdomen black, posteromedial margins and anterolateral corners of ventrites darker. Legs dark brown.

*Head.* Clypeus with moderately dense and shallow punctation consisting of crescent-shaped punctures intermixed with denser, slightly smaller and rather transverse punctures; interstices without microsculpture. Anterior margin of clypeus with a narrow bead. Frontoclypeal suture conspicuous as a zone without punctation, vanishing mesally. Frons with punctation similar to that on clypeus, punctures sparser on sides; interstices without microsculpture. Eyes rather small; interocular distance about 6× the width of one eye in dorsal view. Labrum membranous, nearly completely concealed under clypeus, only with narrowly exposed sinuate anterior margin. Mentum (Fig. 10a) subtrapezoid, widest at posterior fourth, about 2.1× wider than long, 1.4× wider at widest part than at anterior margin, concave in anterior half, strongly emarginated anteromesally; surface glabrous, punctures rather small, shallow and sparse, almost vanishing anteromesally, interstices without microsculpture. Antenna with 9 antennomeres, scapus ca. 1.9× as long antennomeres 2–6 combined; antennal club moderately elongate, about twice as long as wide, about as 1.2× as long as scapus; antennomere 9 acuminate at apex.

*Prothorax.* Pronotum transverse, widest at base 2.1–2.2× wider than long; 1.6–1.7× wider at base than between front angles, 1.7× wider than head including eyes, as convex as the elytra in lateral view. Punctation (Fig. 10b) moderately dense and shallow, consisting of crescent-shaped punctures intermixed with denser, slightly smaller and rather transverse punctures; punctures slightly feebler on sides. Prosternum strongly tectiform medially, medial ridge (Fig. 10c) weakly thickened anteriad, forming a small rounded to slightly pointed process. Antennal grooves distinct, with lateral margin curved, slightly feebler anteriad.

*Pterothorax*. Scutellar shield 1.25× as long as wide, sparsely punctured. Elytra widest at anterior fifth, 2.7–3.0× as long as pronotum, 1.2× as wide as pronotum, surface (Fig. 10d) glabrous, with 10 series of punctures; series 6, 8 and 9 not reaching elytral base, serial punctures of same size in all series; intervals moderately convex; interval punctation composed by crescent-shaped punctures intermixed with denser, slightly smaller and rather transverse punctures in all intervals. Humeral bulge indistinct. Mesoventral plate (Fig. 10f) narrowly elongate, ca. 5.8× as long as wide, widest in anterior two-fifths, more strongly narrowing towards anterior apex which is pointed, posterior tip rounded, slightly overlapping over anterior portion of metaventrite; surface with a few sparse coarse punctures. Metaventrite (Fig. 10g) with narrow raised pentagonal area, 1.2× longer than wide, glabrous, weakly and sparsely punctuate, punctures with fine setae at least along margins of the elevation; bare elevated area reaching anterior margin of metaventrite; punctures absent at two slightly elongate areas in the center; femoral lines absent; lateral parts of metaventrite densely covered by short pubescence.

*Legs.* Femora with sparse rather shallow punctures ventrally, interstices with weak microsculpture consisting of longitudinal lines; tibial grooves distinct. Tibiae with rather small lateral spines. Metatibiae moderately narrow and elongate, straight, 0.3–0.4× as long as elytra, 5.3× as long as wide. Metatarsus long, 0.9× as long as metatibia, with short rather stout setae ventrally.

*Abdomen.* With five ventrites, first abdominal ventrite (Fig. 10h) about as long as the second and third ventrites combined, with distinct median longitudinal carina narrowing posteriad, not projecting posteriorly in both sexes; fifth ventrite with acuminate apex, weakly bulged in males and with a triangularly bulged apical projection in females (Fig. 10i).

*Genitalia.* Sternite 9 (Fig. 5p) asymmetrical basally, median process narrow, ca. as long as lateral struts, acuminate at apex, without subapical setae. Phallobase (Fig. 5m) almost twice as long as parameres, asymmetrically narrowing at base, base narrowly rounded and hooked. Parameres continuously narrowing apically, apex pointed, with two minute setae. Median lobe (Fig. 5n) widest in apical third, slightly narrowing towards base, continuously narrowing in apical third; apex (Fig. 5o) acuminate with rounded tip, apical part with numerous spines directed backwards; gonopore moderately large, subapical; basal portion with dorsal plate narrow and simply bifid basally.

###### Etymology.

We dedicate this species to the eminent physicist and chemist Marie Skłodowska-Curie, on whose honor the Marie Skłodowska-Curie actions program of the European Union, funding this research, is named.

###### Distribution.

Jamaica: Saint Thomas (Fig. 15b).

###### Biology.

The specimens were collected on dung and using dung and carrion-baited traps in cloud forests.

##### Cercyon floridanus

Taxon classificationAnimaliaColeopteraHydrophilidae

Horn, 1890

Figures 2d–f , 7a–d , 16a

Cercyon floridanus Horn, 1890: 303.
Cercyon floridanuus Smetana (Thomas et al. 2013: 33 *lapsus calami*)

###### Figures in Flickr.

www.flickr.com/photos/142655814@N07/albums/72157676249653654

###### Type locality.

Florida (without specific locality).

###### Greater Antillean specimens studied.

**CAYMAN ISLANDS: Grand Cayman**: blacklight trap, vi.1992, leg. blacklight trap (1 male; FSCA), blacklight trap, vi.1992, lgt. P. Fitzgerald (1: FSCA); Queen Elizabeth Botanic Garden, blacklight trap, 28.v.2009, leg. Thomas, Turnbow & Ball (1: FSCA); Georgetown, blacklight, 30.iii.1973. E.J. Gerberg (1: FSCA).

###### Published Greater Antillean records.

**GRAND CAYMAN**: 3 km W Colliers, 19°21'N, 81°07'W (Thomas et al. 2013).

###### Diagnosis.

Body size 2.35–2.70 mm; dorsal surface of head (Fig. 2d) completely black, pronotum black sometimes with undefined piceous areas at lateral margins; elytra black (Figs 2d, f), with large rather sharply-defined yellowish to reddish-yellow lateroapical areas reaching apex lateralmost interval, medial ridge of prosternum anteriorly very weakly projected ventrally; mesoventral plate (Fig. 7d) very wide, ca. 1.9× as long as wide; metaventrite (Fig. 7d) without femoral lines, with broad raised pentagonal area (about 0.67× as long as wide) with large, deep and semicircular punctures; first abdominal ventrite without spiniform process in both sexes; apex of fifth ventrite without triangularly bulged projection at apex in both sexes; aedeagus (Fig. 7a) with parameres slightly shorter than phallobase, sinuately widened and bearing long setae at apex; median lobe widest at midlegth, narrowing to very finely truncate apex, without spines. For complete description see Smetana (1978).

*Cercyon floridanus* is part of the *C. tristis* group according to Smetana (1978) It resembles members of the *C. gimmeli* species group, *C. sklodowskae* sp. n. and *C. praetextatus*. Besides of the features of the aedeagus (Fig. 7a–c), it can be easily distinguished from them by the distinctly by the smallersize (2.35–2.70 mm), wider mesoventral plate (1.9× as long as wide in *C. floridanus*, 3.3–5.8× as long as wide in the other species). Besides that, females of *C. floridanus* lack the triangular projection on the apex of the fifth abdominal ventrite (present in *C. sklodowskae*), has a very small process of mid-prosternal ridge (large in *C. gimmeli* species group), and almost straight metatibia (curved in *C. gimmeli* species group).

###### Distribution.

*Cercyon floridanus* is distributed in the southeastern USA, mainly in Florida, but rare records are also known from Georgia, Louisiana and Mississippi (Smetana 1978). In the Greater Antilles it is only known from Cayman Islands, from where it was first reported by Thomas et al. (2013) under the name “*C. floridanuus* Smetana” (Fig. 16a).

##### Cercyon praetextatus

Taxon classificationAnimaliaColeopteraHydrophilidae

(Say, 1825)

Figures 2g–i , 6a–d , 13a–b , 15c

Sphaeridium praetextatum Say, 1825: 190.
Cercyon praetextatum (Say): Melsheimer, 1853: 37. For complete synonymy see Smetana (1978: 84) and Hansen (1999: 286).

###### Figures in Flickr.

www.flickr.com/photos/142655814@N07/albums/72157669492876764

###### Type locality.

USA, “Cambridge” (based on neotype designated by Smetana 1978).

**Greater Antillean specimens studied. CUBA: Santiago de Cuba**: Dos Caminos, farm field, MV lights, 20.18043°N, 75.77806°W, 165 m, 23.iii.2013, leg. A. Smith & A. Deler-Hernández (1 spec.: NMPC). **Cienfuegos**: Cumanayagua municipality, JBC [= Jardín Botánico de Cienfuegos], Soledad, 22°7'18.44"N, 80°19'35.26"W, 3.x.2012, leg. A. Deler-Hernández (1 spec.: NMPC). **CAYMAN ISLANDS: Grand Cayman**: black-light trap, 17.v.1992, leg. P. Fitzgerald (1 spec.: FSCA). **DOMINICAN REPUBLIC: La Vega Prov.**: Jarabacoa, 440 m a.s.l., riverside, UV light, 24.vii.-2.viii.1995, leg. S. & J. Peck (3 spec.: CMN). La Ciénega de Manabao, Park Headquarters, 915 m a.s.l. black-light, 3-5.vii.1999, leg. R.E. Woodruff (1 spec.: FSCA).

###### Published Greater Antillean records.

**CUBA: Habana Province**: Laguna de Ariguanabo (Spangler 1981). **JAMAICA**: without precise locality (Smetana 1978).

###### Diagnosis.

Body size 2.7–4.1 mm; dorsal surface of head (Fig. 2g) black, with a pair of small reddish-brown spots on vertex, sometimes fused to one spot; pronotum black with sharply defined yellowish to reddish areas at anterolateral corners, sometimes extending to complete lateral margins; elytra black, with large sharply-defined yellowish to reddish-yellow lateroapical area reaching about apical fourth, laterally reaching elytral base in lateralmost interval, yellow spot not extended to humeral area basally; medial ridge of prosternum anteriorly forming a small rounded to slightly pointed process; mesoventral plate (Fig. 13a) wide, ca. 3.3× as long as wide; metaventrite (Fig. 13b) without femoral lines, with narrow raised pentagonal area (ca. as long as wide); first abdominal ventrite without spiniform process in both sexes; apex of fifth ventrite without triangularly bulged projection at apex in both sexes; aedeagus (Fig. 6a–c) with parameres almost twice as long as phallobase, sinuately widened and bearing long setae at apex; median lobe widest at midlegth, narrowing to pointed apex, without spines. For complete description see Smetana (1978).

This species was assigned to the *C. marinus* group according to Smetana (1978). By the coloration of pronotum and elytra (Fig. 2g, i), *C. praetextatus* may be confused with members of the *C. gimmeli* species group and with *C. sklodowskae* sp. n. Besides of the features of the aedeagus (Fig. 6a–c), it can be easily distinguished from them by the distinctly wider mesoventral plate (3.3× as long as wide in *C. praetextatus*, 5.7–5.8× as long as wide in the other species) and the yellow stripe along lateral margin of elytra not expanding basally. Besides of that, females of *C. praetextatus* lack the triangular projection on the apex of the fifth abdominal ventrite (present in *C. sklodowskae*), has a very small process of mid-prosternal ridge (large in *C. gimmeli* species group), and almost straight metatibia (curved in *C. gimmeli* species group).

###### Distribution.

*Cercyon praetextatus* is widely distributed in North America (southern Canada, USA, Mexico; Smetana 1978; Ryndevich 2004) and reaches to Central America (Guatemala, Costa Rica; Smetana 1978) and to the Caribbean (Cayman Islands, Cuba, Dominican Republic, Jamaica, Smetana 1978, Spangler 1981, this paper); it has also been introduced to Argentina (Fikáček 2009). We report it here from Dominican Republic (La Vega Province) and the Cayman Islands for the first time (Fig. 15c).

###### Biology.

This species seems to prefer wet environments, living primarily on many kind of organic debris, like decomposing plant remnants, carrion and dung (Smetana 1978). In Cuba and the Dominican Republic this species has been attracted to light.

##### Cercyon spiniventris sp. n.

Taxon classificationAnimaliaColeopteraHydrophilidae

http://zoobank.org/B172C05C-D9C0-4863-AF24-EB754A45BFC6

**Figure 3.**
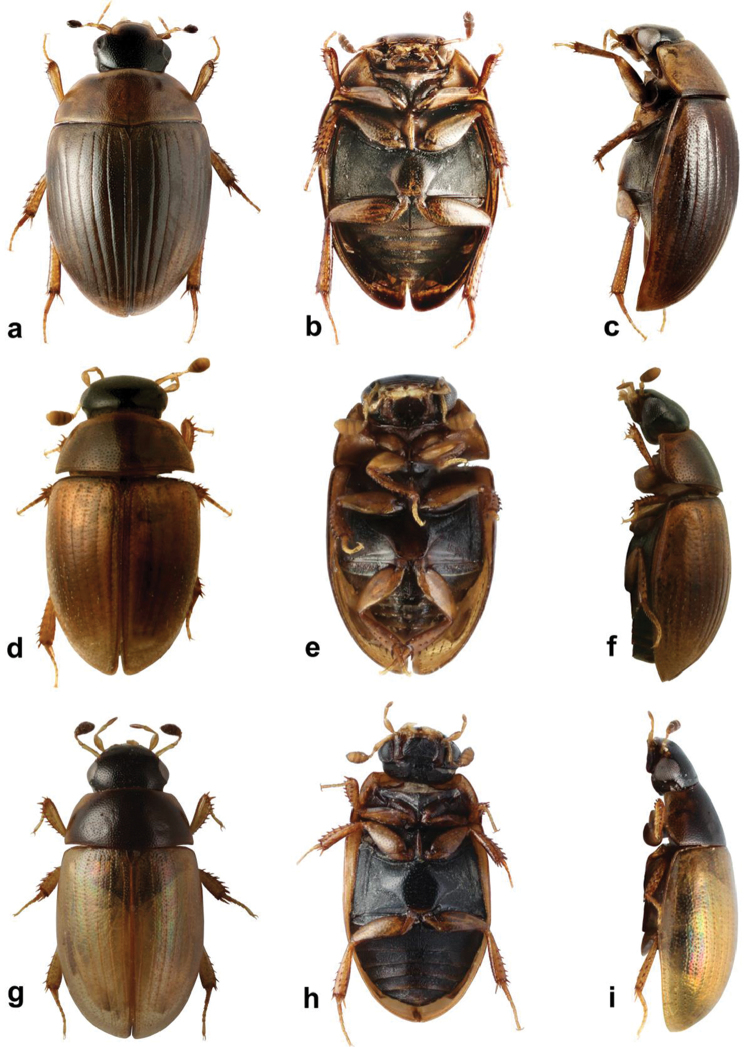
*Cercyon* spp. **a–c** *Cercyon spiniventris* sp. n. **d–f** *Cercyon nigriceps* Marsham **g–i** *Cercyon quisquilius* Linnaeus **a, d, g** dorsal habitus **b, e, h** ventral habitus **c, f, i** lateral habitus.

Figures 3a–c , 6e–h , 11a–i , 16a

###### DNA barcode.

GANTC010-17

###### BIN ID.

BOLD:ADF5572

**Figures in Flickr**: www.flickr.com/photos/142655814@N07/albums/72157671689463811

###### Type locality.

Dominican Republic, Monseñor Nouel Province, Parque Nacional La Humeadora; 11.6 km SSW of Piedra Blanca, 636 m a.s.l., 18°44.92'N, 70°21.63'W.

###### Type material.

**Holotype** (male): “DOMINICAN REP.: Msñ. Nouel, PN La Humeadora; 11.6km SSW, of Piedra Blanca; 18°44.92'N, 70°21.63'W; 636 m; 8.ix.2014, Deler, Fikáček, Gimmel DR41 // in horse excrement in moist broad-leaf forest in a valley of a small stony stream” (NMPC) [DNA extract: MF1216.1]. **Paratypes**: **DOMINICAN REPUBLIC: Barahona**: “DOMINICAN REP.: Prov. Barahona. nr. Filipinas, Larimar Mine: 26-VI/7-VII-1992: Woodruff, Skelley, Skillman. dung trap (1 males: FSCA). “DOMINICAN REP.: Prov. Barahona. nr. Filipinas, Larimar Mine: 26-VI/7-VII-1992: Woodruff & Skelley, rat carrion” (2 females: FSCA). **Monseñor Nouel**: same data as the holotype (7 males, 3 females, 12 spec.: NMPC; 2 males: BCPC; 2 males: BMNH; 2 males: CNC; 3 males: CNIN; 3 males: NHMW; 4 males, 1 female: SBNM; 3 males: SBP; 2 ZMUC). **Duarte**: “DOMINICAN REP.: Duarte. 9.1 km SW of El Factor, slope above La Factoria; 19°15.30'N, 69°56.52'W; 255 m; 4.ix.2014. Deler, Fikáček, Gimmel DR32 // area with cocoa plantations and small remnants of forests at very steep slopes: in horse excrement” (2 males: NMPC). **Independencia**: “DOMINICAN R.: Independencia, PN Sierra de Neiba, 11.3 km NW of La Descubierta; 1650 m, 18°39.81'N, 71°46.17'W; 18.viii.2014, Deler,Gimmel DR13 // disturbed montane cloud forest with many ferns and mosses: in cow excrement” (2 males, 4 females, 1 spec.: NMPC). **La Vega**: “DOMINICAN REP.: La Vega, PN A. Bermúdez, 8 km W of Manabao, 19°4.05'N, 70°51.98'W, 1140 m, 22-26.viii.2014, Deler, Fikáček, Gimmel DR16 // montane broad-leaf forest: in cow and horse excrement” (12 males, 15 females: NMPC; 1 male: BMNH; 1 male: CNIN; 2 males: FSCA; 1 male: MNHNSD; 3 males, 2 females: SBNM) [DNA extraction of one male: MF1753 in NMPC]; “DOM. REP; La Vega Prov., 10km NE Jarabacoa, Hotel Montana, forest, 18.VII-4.VIII.95, 550m, FIT, S.+J. Peck, 95-30” (2 females: CMN); “DOM. REP; La Vega Prov., PN. A. Bermudez, Cienaga, 19.VII-2.VIII.95, 1000m, trop.evgrn.for., FIT, S.+J. Peck, 95-32” (2 females: CMN); “DOM. REP; La Vega Prov., PN. A. Bermudez, Cienaga, 19.VII-2.VIII.95, 1020m, trop.evgrn.for., FIT, S.+J. Peck, 95-34” (1 male: CMN); “DOM. REP; La Vega Prov., PN. A. Bermudez, Cienaga, 21.-24.VII.95, 1000m, for. carrion trap, S.+J. Peck, 95-38” (1 female: CMN). **Samaná**: “DOMINICAN REP.: Samaná, MN Salto El Limón 2.8 km SSW of El Limón; 19°16.56'S 69°26.47'W; 160 m; 2.ix.2014, Deler, Fikáček, Gimmel. DR29a // secondary vegetation and tiny remnants of forest among coffee plantations and pastures: in horse excrement” (3 males, 3 females: NMPC).

###### Diagnosis.

Body size 3.4–4.1 mm; dorsal surface of head black with yellowish anterolateral margins of clypeus (Fig. 3a); pronotum homogeneously light brown, elytra greyish-brown; medial ridge of prosternum anteriorly with a small rounded process (Fig. 11c); mesoventral plate narrow, ca. 5.9× as long as wide; metaventrite (Fig. 11f) without femoral lines; raised pentagonal area of metaventrite moderately wide, 0.9× as long as wide; first abdominal ventrite with an spiniform process in females (Fig. 11h), without process in males; apex of fifth abdominal ventrite without apical triangular projection in both sexes (Fig. 11i); aedeagus with parameres about as long as phallobase (Fig. 6e), median lobe narrowly parallel-sided, acute at apex, without spines.

*Cercyon spiniventris* somewhat resembles *C. nigriceps* by the dorsal coloration pattern (predominantly black head and rather homogeneously brown pronotum and elytra); it can be easily distinguished from *C. nigriceps* by much larger body size (3.4–4.1 mm in *C. spiniventris*, 1.0–2.1 mm in *C. nigriceps*) and by the lack of femoral lines on the metaventrite (present in *C. nigriceps*). *Cercyon spiniventris* is unique among Caribbean *Cercyon* species by a presence of a long spiniform process in the first abdominal ventrite of females.

###### Description.

*Body* (Fig. 3a–c). 3.4–4.0 mm long (length of holotype: 3.5 mm); moderately short-oval, 1.8–1.9× as long as wide, widest at basal fourth of elytra; moderately convex, 2.9–3.0× as long as high (height of holotype: 1.15 mm). *Coloration.* Dorsal surface of head blackish to pitchy black, clypeus with wide rather sharply defined yellowish area along anterolateral margins, broader at sides. Antennae and ventral surface of head black, mentum with posterior half yellowish brown, mouthparts and antenna yellowish brown, antennal club dark-brown. Pronotum light brown. Prosternum yellowish-brown with posterior half black, hypomeron brown with large black marks on posterior third, and close to the yellowish-brown lateral margins. Elytra dark greyish-brown, with lateral and anterior margins, apex and epipleura slightly paler. Ventral surface of mesothorax blackish to pitch-black, with procoxal rests and mesoventral plate brown. Metepisternum black. Metaventrite black with paler raised anteromedial part. Abdomen black, posteromedial margins and anterolateral corners of ventrites brownish. Legs brown, femora dorsally black.

*Head.* Clypeus with dense and moderately deep punctation consisting of crescent-shaped setiferous punctures intermixed with denser, smaller and rather transverse non-setiferous punctures; interstices without microsculpture. Anterior margin of clypeus with a narrow bead. Frontoclypeal suture conspicuous as a zone without punctuation, vanished mesally. Frons with punctation similar to that on clypeus, punctures of same shape all over; interstices without microsculpture. Eyes rather small; interocular distance about 5.4× the width of one eye in dorsal view. Labrum membranous, nearly completely concealed under clypeus, only with narrowly exposed sinuate anterior margin. Mentum (Fig. 11a) subtrapezoid, widest at posterior fourth, about 2× wider than long, 1.5× wider at widest part than at anterior margin, weakly concave in anterior half; surface glabrous, punctures large and deep, becoming coarser anteromesally, interstices on anterior half with transverse depressions near each puncture. Antenna with 9 antennomeres, scapus ca. 1.8× as long as antennomeres 2–6 combined; antennal club moderately elongate, about twice as long as wide, about as 1.2× as long as scapus; antennomere 9 acuminate at apex.

*Prothorax.* Pronotum transverse, widest at base 2.1–2.3× wider than long; 1.7× wider at base than between anterior angles, 1.8× wider than head including eyes, as convex as elytra in lateral view. Punctation rather dense and moderately deep, consisting of crescent-shaped setiferous punctures intermixed with denser, smaller and rather transverse non-setiferous punctures; punctures slightly feebler on sides. Prosternum (Fig. 11b–c) strongly tectiform medially, medial ridge very weakly thickened anteriad, forming a small rounded process. Antennal grooves distinct, with lateral margin curved, feebler anteriad.

*Pterothorax*. Scutellar shield about as long as wide, moderately densely punctured. Elytra widest at anterior fifth, 2.7–2.9× as long as pronotum, 1.1–1.2× as wide as pronotum, surface (Fig. 10d) glabrous, with 10 series of punctures; series 6, 8 and 9 not reaching elytral base, serial punctures of same size in all series; intervals moderately convex; interval punctation composed of crescent-shaped setiferous punctures intermixed with denser, smaller and rather tranverse non-setiferous punctures; setiferous punctures present on all intervals; interstices without microsculpture. Humeral bulge indistinct. Mesoventral plate (Fig. 11f) narrowly elongate, ca. 5.9× as long as wide, widest at midlength, gradually and symmetrically narrowing to pointed apices, posterior tip slightly overlapping over anterior part of metaventrite; surface with coarse punctures. Metaventrite (Fig. 11g) without femoral lines, raised pentagonal area wide, 0.8× as long as wide at widest portion, glabrous, rather weakly and sparsely punctate, punctures with fine setae at least along margins of elevation, punctures absent at two slightly elongate areas in the center, bare area not reaching anterior margin of metaventrite mesally; lateral parts of metaventrite densely covered by short pubescence.

*Legs.* Femora with sparse rather shallow punctures ventrally, interstices with weak granulose microsculpture; tibial grooves distinct. Tibiae with moderately large lateral spines. Metatibiae moderately narrow and elongate, slightly bent outwards, 0.4× as long as elytra, 6.0× as long as wide. Metatarsus moderately long, 0.7–0.8× as long as metatibia, with short rather stout setae ventrally.

*Abdomen* with five ventrites, first abdominal ventrite longer than second and third ventrites combined, with long setae in medial third, median longitudinal carina present, slightly narrowing posteriad, not projecting posteriorly in males, projecting posteriad as a short spine in females (Fig. 11h); ventrite 5 with acuminate apex in both sexes.

*Genitalia.* Median projection of sternite 9 (Fig. 6h) rounded apically, without subapical setae, median portion narrowing posteriorly, shorter than lateral struts. Phallobase (Fig. 6e) about as long as parameres, asymmetrically narrowing basally, base acuminate and slightly hooked. Parameres weakly narrowing apically, subsinuate near apex, apex pointed apically. Median lobe (Fig. 6f) narrow, parallel-sided throughout, apex acuminate, gonopore moderately large, situated subapically; basal portion with dorsal horseshoe-shaped plate, base bifid. throughout, apex acuminate, gonopore moderately large, situated subapically; basal portion with dorsal horseshoe-shaped plate, base bifid.

###### Etymology.

The name of this species is derived from Latin words *spina* (spine) and *venter* (underside), in reference to the spine-like process on the first abdominal ventrite of females.

###### Distribution.

Dominican Republic: Duarte, Independencia, La Vega, Monseñor Nouel, Samaná (Fig. 16a).

###### Bionomics.

Most of the specimens were collected in cow and horse dung in tropical forest and surrounding pastures.

##### Cercyon nigriceps

Taxon classificationAnimaliaColeopteraHydrophilidae

(Marsham, 1802)

Figures 3d–f , 6i–k , 13c–d

Dermestes nigriceps Marsham, 1802: 72.
Cercyon nigriceps Stephens (1829: 151). = Dermestes atricapillus Marsham, 1802: 72 (synonymized by Gemminger and Harold 1868: 498; precedence of C. nigriceps over C. atricapillus determined by Stephens 1939: 97, see also Hansen 1999: 284). = Cercyon striatus Sharp, 1882: 108 (synonymized by Fikáček 2009: 354). = Cercyon panamensis Hansen, 1999: 286 (replacement name of C. striatus Sharp; synonymized by Fikáček 2009: 354). For complete synonymy see Smetana (1978) and Hansen (1999).

###### DNA barcode.

GANTC015-17

###### BIN ID.

BOLD:AAO0116

###### Figures in Flick.

www.flickr.com/photos/142655814@N07/albums/72157671425572500

###### Type locality.

“Britannia” [= Great Britain, without specified locality].

###### Specimens examined.

**CAYMAN ISLANDS: Cayman Brac**: black-light trap, 06.vi.2008, lgt. R.H. Turnbow & B.K. Dozier (3 spec.: FSCA); Agricultural Exp. Sta. S. Of Songbird Dr., black-light trap, 04.vii.2013, leg. M.C. Thomas (3 spec.: FSCA); **CUBA: Cienfuegos**: Cumanayagua municipality, 22°7'18.44"N, 80°19'35.26"W, 722 m, 21.v.2013 (1 spec.: NMPC). **Guantánamo**: El Yunque, 0.5-1.0 km W of Campismo Popular, 20°20.1'N, 74°33.6'W, 40–50 m. 10.vi.2012, leg. Deler-Hernández & Fikáček (MF01) (8 spec.: NMPC). **Holguín**: Mayarí municipality, Feltón, 20°43'7.92"N, 75°37'59.19"W, 23.iii.2013, leg. Deler-Hernández (28: NMPC). **Santiago de Cuba**: El Vivero, 1.6 km E of Dos Caminos, 20°10.8'N, 75°46.4'W, 150 m, 20-21.vi.2012, leg. Deler-Hernández & Fikáček (MF18) (54 spec.: NMPC) [DNA extract: MF604]; San Luis Municipality, Dos Caminos, 20°10'57.82"N, 75°46'40.84"W, leg. Deler-Hernández (16 spec.: NMPC). **Artemisa**: Cañon de Santa Cruz, Río de Santa Cruz, 22°45'1.29"N 83°08'56.36"W, 199 m a.s.l., 16.vii.2016, leg. A. Deler-Hernández (8 spec.: NMPC) [DNA extraction: MF1750]. **DOMINICAN REPUBLIC: Samaná**: Samaná, dam 2.5 km N of Samaná, in older cow excrements dampered by recent rains at the grassy bank of a reservoir, 19°13.70'N, 69°19.85'W, 58 m a.s.l., 5.ix.2014, leg. Deler, Fikáček & Gimmel (DR35) (3 spec.: NMPC); MN Salto El Limón 2.8 km SSW of El Limón, secondary vegetation and tiny remnants of forests among coffee plantations and pastures, cow excrements, 19°16.56'N, 69°26.47'W, 2.ix.2014, leg. Deler-Hernández, Fikáček & Gimmel (DR29a) (1 spec.: NMPC). **La Altagracia**: Nisibon, Black-light trap, 03.v.1978, lgt. R.E. Woodruff & G.B. Fairchild (2 spec.: FSCA); **La Vega**: 7.0 km W of Manabao, side of a stony stream in a valley with scattered houses and plantations surrounded by montane forest, in cow excrement, 19°04.56'N, 70°51.46'W, 1185 m a.s.l., 23.viii.2014, leg. Deler-Hernández, Fikáček & Gimmel (1 spec.: NMPC). **Monseñor Nouel**: PN La Humeadora; 11.6 km SSW, of Piedra Blanca, in horse excrement in moist broad-leaf forest in a valley of a small stony stream, 18°44.92'N, 70°21.63'W, 636 m a.s.l., 8.ix.2014, leg. Deler, Fikáček & Gimmel (DR41) (2 spec.: NMPC). **Monte Cristi**: 8.2 km. N Villa Elisa, 01.vi.1994, leg. R. Turnbow (1 spec.: FSCA). **San Pedro de Macoris**: Juan Dolio, at light, 10.-18.xii.2005, leg. Fencl (15 spec.: NMPC). **HAITI: Artibonite**: Montrouis, black-light trap, 05.vii.1977, leg. J.H. Frank (2 spec.: FSCA). **PUERTO RICO: Naguabo**: El Yunque National Forest (southern part), 3.45 km N of Río Blanco at road PR191, in horse excrements on exposed small pasture on the slope of El Yunque massive, 18°14.8'N, 65°47.9'W, 170 m a.s.l., 24.vi.2016, leg. Deler-Hernández, Fikáček & Seidel (PR2a) (17 spec. NMPC) [DNA extraction of one specimen: MF1732]; El Yunque National Forest (southern part), 4.9 km N iof Río Blanco, margin of the rainforest in an area with many flowering *Etlingera elatior* plants, FIT, 18°15.8'N, 65°47.3'W, 495 m a.s.l., 24.vi.-2.vii.2016, leg. Fikáček & Seidel (PR11)(1 spec.: NMPC). **Arecibo**: small settlement in Bosque Estatal Río Abajo, small settlement in the middle of the lowland forest, horse excrement, 18°19.7'N, 66°42.1'W, 340 m a.s.l., 27.vi.2016, leg. Deler-Hernández, Fikáček & Seidel (PR15) (13 spec.: NMPC). **Cabo Rojo**: Boquerón, black light, 18°13.11'N, 67°10.96'W, 5-6.x.2011, leg. A. Segarra (7 spec.: UPRM). **Rio Grande**: El Verde Biological Station, at light, 26.v.1994, leg. R. Turnbow (2 spec.: FSCA). **Lesser Antilles: ANTIGUA**: Christian valley, blacklight trap, 19.viii.1991, leg. FAO insect survey (1 spec.: SBP); same locality and collector, 26.vii.1991 (1 spec.: SBP); same locality and collector, 29.x.1991 (3 spec.: SBP); same locality and collector, 14.-15.ix.1991 (1 spec.: SBP). **GRENADA**: St. Andrew, Mirabeu Agriculture Lab, light trap, 9.iv.1990, leg. J. Telesford (1 spec.: SBP). **SAINT LUCIA**: Vieux Fort, horse dung sifting, 13°43.9'N 60°53.9'W, 3 m a.s.l., 12.vii.2007, S & J. Peck (07-60) (2 spec.: SBP); Mon Repos, Fox Grove lnn, UV light, 13°51.8'N, 60°54.4'W, 90 m a.s.l., 8.-18.vii.2007, leg. S. & J. Peck (07-50) (1 spec.: SBP). Soufriere, Rechette Pt. 11.VII.1980, leg. L.S. Mahunka (59 spec.: HNHM). **SAINT VINCENT & THE GRENADINES: St. Vincent**: Emerald Valley Hotel E of Layou, horse dung, 13°12.0'N, 61°14.8'W, 20 m a.s.l., 24.viii.2006, S. & J. Peck (06-120) (3 spec.: SBP); same locality, UV light at forest edge, 27.-29.viii.2006, leg. S. & J. Peck (06-123) (1 spec.: SBP). **Union Island**: Chatham Bay, Water Rock Reserve, UV traps in tall forest, 12°36.18'N, 61°26.59'W, 125 m a.s.l.,16.viii.2009, leg. S. Peck (09-64) (2 spec.: SBP); Campbell Miss Irene Reserve, high canopy thorn forest, 12°35.44'N, 61°27.34'W, 85 m a.s.l., 18.viii.2009, leg. S. Peck (09-66) (1 spec.: SBP).

###### Published records from the Caribbean.

**CUBA: Matanzas**: Cárdenas (as *C. centrimaculatum*, Gundlach 1891); without precise locality (as *C. centrimaculatum*, de la Sagra 1857). **JAMAICA**: without precise locality (Leng and Mutchler 1917). **Trelawny**: Good Hope; Duncans (Fikáček 2009). **DOMINICAN REPUBLIC: Pedernales**: 4 km W of Oviedo (Fikáček 2009). **GUADELOUPE**: without precise locality (Leng and Mutchler 1914); Peck et al. 2014). **MONTSERRAT**: without precise locality (Peck et al. 2014).

###### Diagnosis.

Body size 1.0–2.1 mm; dorsal surface of head black; pronotum (Fig. 3d) reddish brown, rarely with a vaguely darker central area; elytra uniformly reddish-brown, rarely with a vaguely darker central area; mesoventral plate (Fig. 13c) narrow, ca. 6× as long as wide; metaventrite (Fig. 13d) with complete femoral lines; first abdominal ventrite without spiniform process of both sexes; apex of fifth ventrite without a triangularly bulged projection in both sexes; aedeagus with parameres twice as long as phallobase (Fig. 6i), median lobe (Fig. 6j) continuously acuminate, with long narrowly acute apex, without spines.

This species was assigned to *C. nigriceps* group (= *C. atricapillus* group) by Smetana (1978). *Cercyon nigriceps* can be distinguished from other species in the region by its small size and the presence of complete femoral lines on the metaventrite. It may be confused with representatives of the genus *Oosternum* by the small body size and coloration, but differs from them by presence of femoral lines (absent in *Oosternum*), absence of anterolateral ridge on mesoventrite (present in all *Oosternum*) and by very narrow mesoventral plate (1.7–2.8× as long as wide in *Oosternum*, see Deler-Hernández et al. 2014).

###### Distribution.

This is an adventive species currently distributed in all zoogeographical regions. In Greater Antilles widespread in all islands: Cayman Islands, Cuba (Artemisa, Cienfuegos, Guantánamo, Holguín, Matanzas, Santiago de Cuba; Gundlach 1891), Dominican Republic (La Altagracias, La Vega, Monte Cristi, Samaná, San Pedro de Macoris; this paper), Haiti (Artibonite, this paper), Jamaica (Trelawny; Fikáček 2009) and Puerto Rico (Naguabo, Arecibo, Cabo Rojo). It is also widespread in the Lesser Antilles (Antigua, Grenada, Saint Lucia, Saint Vincent and the Grenadines; this paper) (Fig. 16b). Based on the record by de la Sagra (1857), the species was introduced to the Greater Antilles no later than the first half of the 19^th^ century.

###### Biology.

A terrestrial species collected in cow and horse dung and in decaying plant matter (e.g., compost piles). It is also frequently collected at light.

##### Cercyon quisquilius

Taxon classificationAnimaliaColeopteraHydrophilidae

(Linnaeus, 1761)

Figures 3g–i , 6l–n , 13e–f , 15c

Scarabaeus quisquilius Linnaeus, 1761: 138.
Cercyon quisquilium Stephens (1829: 153). For complete synonymy see Smetana (1978) and Hansen (1999).

###### Figures in Flickr.

www.flickr.com/photos/142655814@N07/albums/72157671688128241

###### Type locality.

“Suecia” [= Sweden, without specified locality].

###### Specimens examined.

**CUBA: Holguín**: Mayarí Municipality, Feltón, Vuelta Larga, permanent lagoon, 23.iii.2013, leg. A. Deler-Hernández (2 spec.: NMPC) [DNA extraction: MF1599].

###### Published records.

**JAMAICA**: without precise locality (Leng and Mutchler 1917).

###### Diagnosis.

Body size 2.4–3.2 mm; dorsal surface of head completely black; pronotum (Figs 3g, i) black with vaguely defined yellowish to brownish lateral margins, broader in anterolateral corners; scutellar shield black; elytra yellow to brownish-yellow; mesoventral plate (Fig. 13e) narrow, ca. 6.3× as long as wide; metaventrite (Fig. 13f) without femoral lines, with raised pentagonal area very wide, 0.6× as long as wide in widest part; first abdominal ventrite without spiniform process in both sexes; apex of fifth abdominal ventrite without triangularly bulged projection; aedeagus with parameres ca. 0.75× as long as phallobase, narrowing towards slightly lobate apex; median lobe fusiform, without spines.

*Cercyon quisquilius* was assigned to *C. unipunctatus* group according to Smetana (1978). This species can be only confused with *C. nigriceps* in Greater Antilles. It may be distingushed from it by the coloration of the pronotum (blackish with diffuse yellowish areas on lateral margins in *C. quisquilius*, almost homogeneously piceous to reddish brown and similar to elytral coloration in *C. nigriceps*), larger body size (2.4–3.2 mm in *C. quisquilius*, 1.0–2.1 mm in *C. nigriceps*), and by metaventrite without femoral lines and with wide raised median part (with femoral lines and narrower median part in *C. nigriceps*).

###### Distribution.

*Cercyon quisquilius* is a species native to the Palearctic Region, but currently introduced to the Nearctic, Neotropical and Australian Regions (Smetana 1978; Hansen 1999; Fikáček 2009). We are providing the first precise records of this species from the Caribbean based of specimens from Cuba (Holguín province) (Fig. 15c).

##### Cercyon insularis

Taxon classificationAnimaliaColeopteraHydrophilidae

Chevrolat, 1863

**Figure 4.**
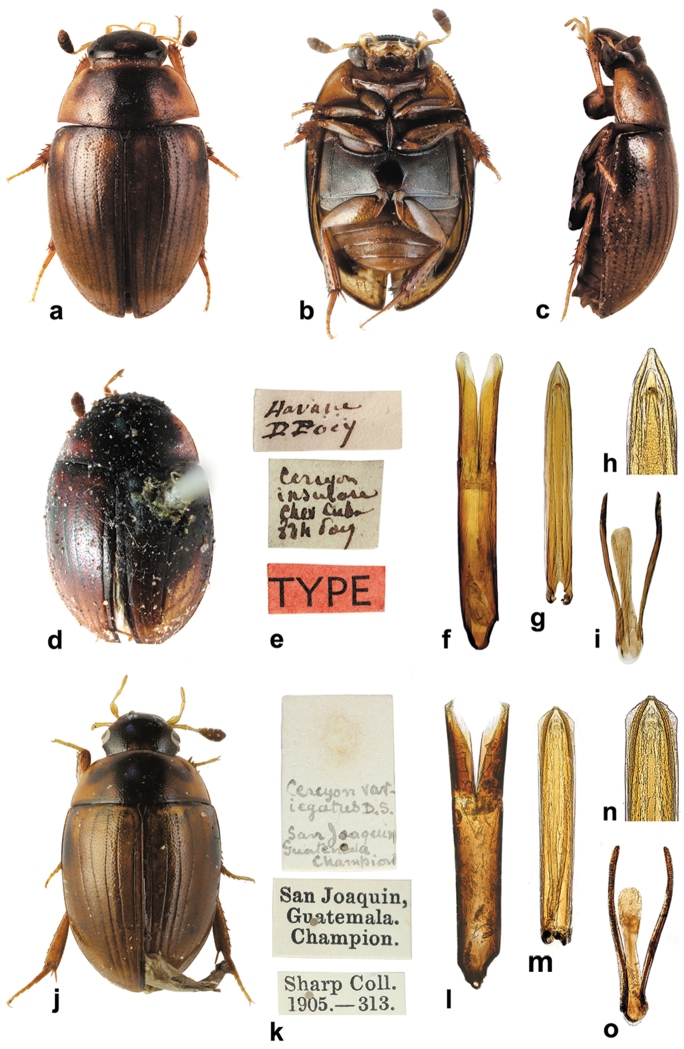
*Cercyon insularis* Chevrolat and *C. variegatus* Sharp. **a–i** *C. insularis*: **a–c** dorsal, ventral and lateral habitus of the non-type specimen from Cuba **d** habitus of the holotype **e** labels of the holotype **f–i** male genitalia of non-type specimen from Dominican Republic. **j–o** lectotype of *C. variegatus*: **j** dorsal habitus **k** labels **l–o** male genitalia. Genital parts illustrated: **f, l** tegmen of aedeagus **g, m** median lobe of aedeagus **h, n** detail of apex of median lobe; **i, o** 9th sternite.

Figures 4a–i , 12 a–i , 16c

Cercyon insulare Chevrolat, 1863: 208.
Cercyon insulare Gundlach (1891: 50, redescription).

###### DNA barcodes.

GANTC001-16, GANTC011-17, GANTC012-17

###### BIN ID.

BOLD:ADC9388.

###### Figures in Flickr.

www.flickr.com/photos/142655814@N07/albums/72157669492393134

###### Type locality.

Cuba: Havana.

###### Type material.

**Holotype** (unsexed specimen): “Havana, D. Poey // Cercyon insulare, Chev Cuba, [illegible] // TYPE [red label]” (MNHN).

**Figure 5.**
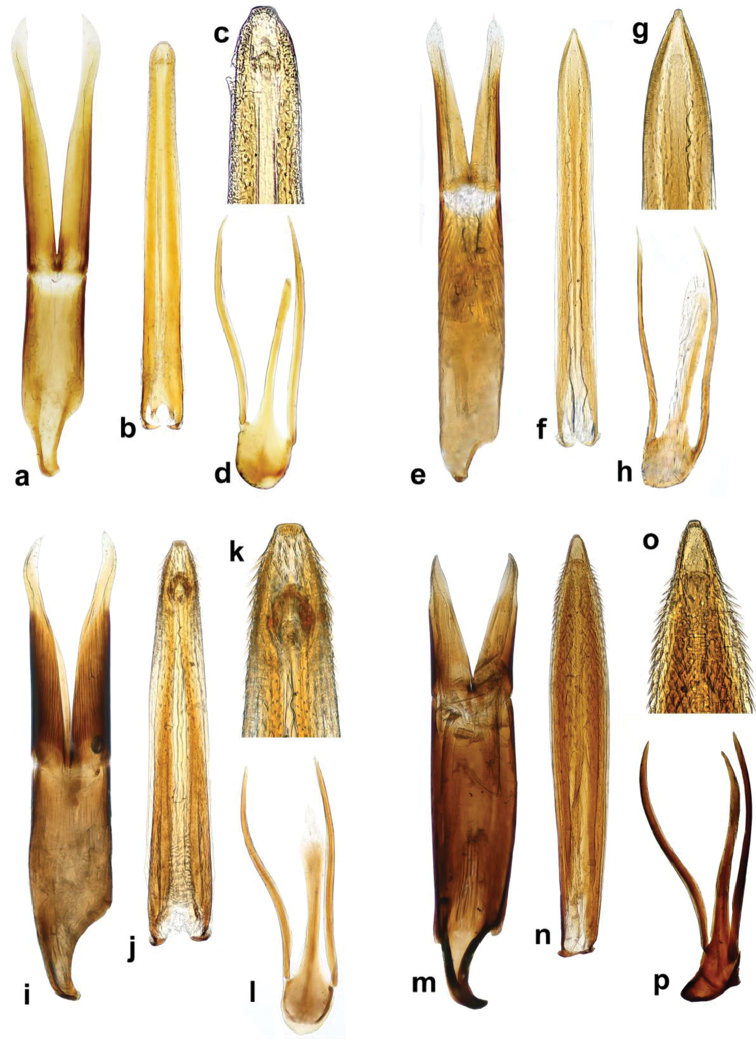
*Cercyon* spp. n. genitalia **a–d** *Cercyon gimmeli* sp. n. **e–h** *Cercyon taino* sp. n. **i–l** *Cercyon armatipenis* sp. n. **m–p** *Cercyon sklodowskae* sp. n. **a, e, i, m** tegmen of aedeagus **b, f, j, n** median lobe of aedeagus **c, g, k, o** 9th sternite.

**Figure 6.**
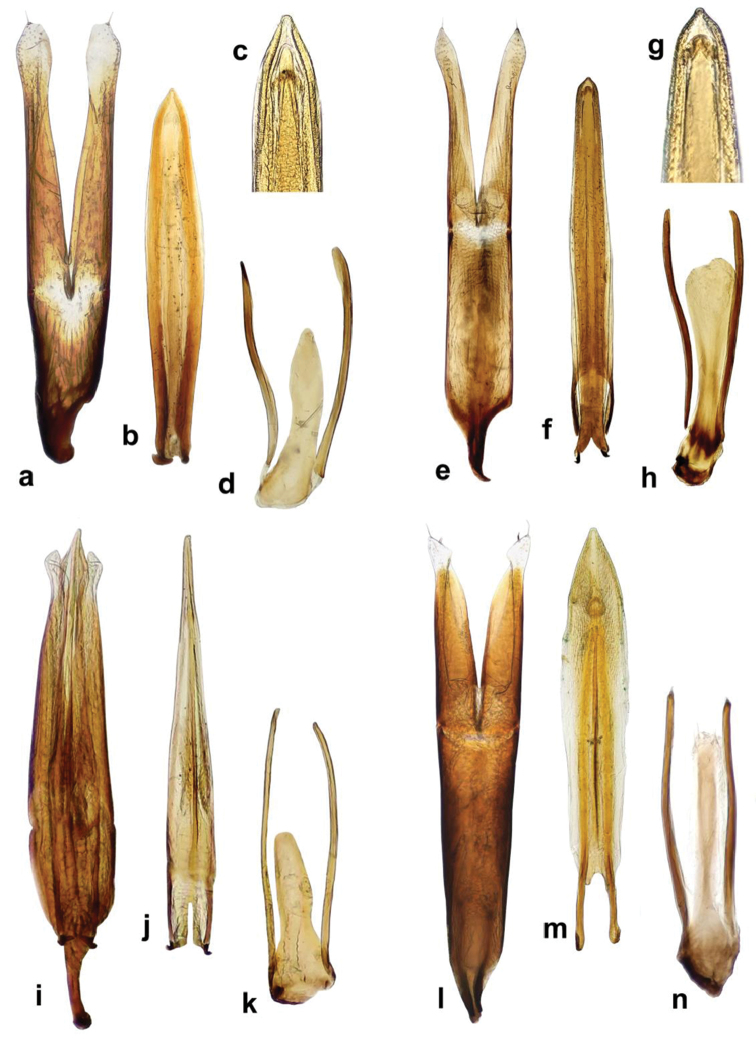
*Cercyon* spp. n. genitalia **a–d** *Cercyon praetextatus* Say **e–h** *Cercyon spiniventris* sp. n. **i–k** *Cercyon nigriceps* Marsham **l–n** *Cercyon quisquilius* Linnaeus **a, e, i, l** tegmen of aedeagus **b, f, j, m** median lobe of aedeagus **c, g** 9th sternite.

**Figure 7.**
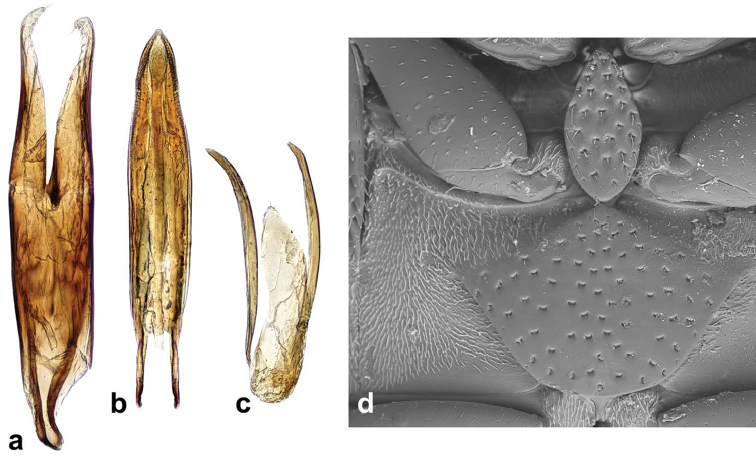
*Cercyon floridanus* Horn **a** tegmen of aedeagus **b** median lobe of aedeagus **c** 9th sternite **d** ventral view of pterothorax.

###### Additional material examined.

**CUBA: Camagüey**: Sierra de Cubitas municipality, Limones-Tuabaquey, 21°35'52.10"N, 77°47'17.62"W, 16.v.2013, leg. R. Anderson (1 spec.: NMPC). **Guantánamo**: El Yunque, 3.2 km SW of campismo popular, at right tributary of Duabe river, secondary evergreen forest, cow excrement, 20°19'N, 74°34'W, 150 m a.s.l., 13.vi.2012, leg. Deler-Hernández & Fikáček (MF09) (7 spec.: NMPC); El Yunque, ca. 1.4 km W of campismo popular, cocoa plantations shaded by palms, cow excrement, 20°20.2'N, 74°33.7'W, 60-150 m a.s.l., 11.vi.2012, Deler-Hernández & Fikáček (MF03) (16 spec.: NMPC). **Santiago de Cuba**: El Vivero, 1.6 km E of Dos Caminos, cow excrements on pasture, 20°10.8'N, 75°46.4'W, 150 m, 20-21.vi.2012, leg. Deler-Hernández & Fikáček (MF18) (1 spec.: NMPC); San Luis Municipality, Dos Caminos, 20°10'57.82"N, 75°46'40.84"W, 3.x.2012, leg. Deler-Hernández (12 spec.: NMPC). **Granma**: PN Turquino, around La Platica, 20°0.7'N, 76°53.4'W, 880 m, 25–26.vi.2012, leg. Deler-Hernández & Fikáček (MF24) (12 spec.: NMPC); PN Turquino, on the trail up to 0.5 km S of La Platica, 20°0.5'N, 76°53.3'W, 920 m, 23–27.vi.2012, leg. Deler-Hernández & Fikáček (MF20) (2 spec.: NMPC); PN Turquino, La Siguapa, ca. 1.5 km SE of La Platica, sifting leaf litter in evergreen forest, 20°0.2'N, 76°52.8'W, 1290 m, 25.vi.2012, leg. F. Cala-Riquelme (MF25) (1 spec.: NMPC) [DNA extract at NMPC] (1: NMPC). **Artemisa**: Cañon de Santa Cruz, Río de Santa Cruz, 22°45'1.29"N 83°08'56.36"W, 199 m a.s.l., 16.vii.2016, leg. A. Deler-Hernández (1 spec.: NMPC) [DNA extraction: MF1749]. **DOMINICAN REPUBLIC: La Vega**: 7.0 km W of Manabao, side of a stony stream in a valley with scattered houses and plantations surrounded by montane forest, in cow excrements, 19°4.56'N, 70°51.46'W, 1185 m a.s.l., 23.viii.2014, leg. Deler-Hernández, Fikáček & Gimmel (DR18) (3 spec.: NMPC); at S margin of Manabao, 19°3.85'N, 70°47.61'W, 912 m a.s.l., 27.viii.2014, leg. Deler-Hernández & Fikáček (DR23) (3 spec.: NMPC). **Samaná**: MN Salto El Limón 2.8 km SSW of El Limón, secondary vegetation and tiny remnants of forests among coffee plantations and pastures, cow excrements, 19°16.56'N, 69°26.47'W, 2.ix.2014, leg. Deler-Hernández, Fikáček & Gimmel (DR29a) (16 spec.: NMPC). **Monseñor Nouel**: PN La Humeadora; 11.6 km SSW, of Piedra Blanca, in horse excrement in moist broad-leaf forest in a valley of a small stony stream, 18°44.92'N, 70°21.63'W, 636 m a.s.l., 8.ix.2014, leg. Deler, Fikáček & Gimmel (DR41) (4 spec.: NMPC) [DNA extracts: MF1214.1, MF1214.2]. **PUERTO RICO: Naguabo**: El Yunque National Forest (southern part), 3.45 km N of Río Blanco at road PR191, in horse excrements on exposed small pasture on the slope of El Yunque massive, 18°14.8'N, 65°47.9'W, 170 m a.s.l., 24.vi.2016, leg. Deler-Hernández, Fikáček & Seidel (PR2a) (19 spec.: NMPC) [DNA extraction: MF1731]; El Yunque National Forest (southern part), 4.9 km N iof Río Blanco, margin of the rainforest in an area with many flowering *Etlingera elatior* plants, FIT, 18°15.8'N, 65°47.3'W, 495 m a.s.l., 24.vi.–2.vii.2016, leg. Fikáček & Seidel (PR11) (3 spec.: NMPC). **Arecibo: s**mall settlement in Bosque Estatal Río Abajo, in the middle of the lowland forest, horse excrement, 18°19.7'N, 66°42.1'W, 340 m a.s.l., 27.vi.2016, leg. Deler-Hernández, Fikáček & Seidel (PR15) (1 spec.: NMPC). **Lesser Antilles: DOMINICA**: Springfield Estate, mature secondary forest, FIT, 15°20.796'N, 61°22.142'W, 30.v.-16.vi.2004, S. & J. Peck (04-86) (3 spec.: SBP). **GRENADA**: Grand Etang Forest Reserve, FIT in rainforest, 12°04.846'N 61°42.333'W, 360 m a.s.l., leg. S. Peck (10-61) (2 spec.: SBP); Grand Etang Forest Reserve, FIT in rainforest, 12°04.162'N 61°42.162'W, 400 m a.s.l., leg. S. Peck (10-63) (2 spec.: SBP); St. Andrew, Mirabeu Agriculture Lab, light trap, 19.iv.1990, leg. J. Telesford (2 spec.: SBP). **SAINT LUCIA**: Mon Repos, 6.5 km W Fox Grove Inn, submontane forest, carrion traps, 13°52.5'N, 60°56.4'W, 300 m a.s.l., leg. S. & J. Peck (0759) (2 spec.: SBP).

###### Published records

**(as *C. variegatus*). JAMAICA**: without precise locality (Smetana 1978). **PUERTO RICO**: without precise locality (Smetana 1978). **DOMINICA**: without precise locality (Leng and Mutchler 1917; Peck 2006).

###### Diagnosis.

Body size 2.4–3.4 mm; dorsal surface of head (Fig. 4a) black with a pair of small pale spots on vertex (sometimes fused in one central spot), pronotum yellowish to dark reddish-brown with a large blackish central spot and two small round blackish spots at sides (sometimes obscured); elytra yellowish with black humeral spot, in dark specimens whole elytral base and suture darkened; medial ridge of prosternum not projected ventrally (Fig. 12c); mesoventral plate narrow, 5.8× as long as wide (Fig. 12f); metaventrite (Fig. 12g) without femoral lines, with raised pentagonal area as long as wide; first abdominal ventrite without spine-like process in both sexes (Fig. 12h); apex of fifth abdominal ventrite (Fig. 12i) without apical projection in both sexes; aedeagus narrow, parameres 0.7× as long as phallobase (Fig. 4f), rounded at apex; median lobe (Fig. 4g) subparallel throughout except for acuminate apex, without subapical spines.

###### Redescription.

*Body.* (Fig. 4a–d) 2.4–3.4 mm long (length of holotype: 2.8 mm); moderately elongate oval, 1.7–1.8× as long as wide, widest at basal fifth of elytra; moderately convex, 2.6–2.8× as long as high (height of holotype: 1.0 mm). *Coloration*. Dorsal surface of head black with a pair of small rufotestaceous spots on vertex. Antennal scape and flagellum and ventral surface of head including mouthparts light-brown, antennal club and mentum dark brown. Pronotum yellowish to dark reddish-brown, with a large blackish central spot and two small round blackish spots at its sides, sometimes connected with central spot. Prosternum yellowish to light brown, hypomeron slightly darkened. Elytra with elongate blackish spot posterior to humeri, elytral base and suture darkened, elytral epipleura uniformly pale. Ventral surface of mesothorax blackish. Metepisternum brown. Metaventrite blackish, darker at medial elevation. Abdomen yellowish to reddish-brown. Legs yellowish to light brown.

*Head.* Clypeus with moderately dense and shallow punctation consisting of small transverse punctures; interstices without microsculpture. Anterior margin of clypeus with narrow bead. Frontoclypeal suture conspicuous as a zone without punctuation, vanished mesally. Frons with punctation similar to that on clypeus, punctures sparser on sides; interstices without microsculpture. Eyes rather small, interocular distance about 6× the width of one eye in dorsal view. Labrum membranous, nearly completely concealed under clypeus, only with narrowly exposed sinuate anterior margin. Mentum (Fig. 12a) subtrapezoid, widest at posterior fourth, about 2× wider than long, 1.5× wider at widest part than at anterior margin, strongly concave in anterior half, anterior margin not emarginate; surface almost glabrous, punctures small, shallow and sparse, almost vanishing anteromesally, interstices without microsculpture. Antenna with 9 antennomeres, scapus ca. 1.8× as long antennomeres 2–6 combined; antennal club moderately elongate, about twice as long as wide, as long as scapus; antennomere 9 acuminate at apex.

*Prothorax.* Pronotum transverse, widest at base 2.1–2.2× wider than long; 1.7–1.8× wider at base than between front angles, 1.8× wider than head including eyes, as convex as pronotum in lateral view. Punctation rather dense and moderately deep, consisting of crescent-shaped punctures intermixed with denser, slightly smaller and rather transverse punctures; punctures slightly feebler on sides. Prosternum (Fig. 12b) strongly tectiform medially, median ridge (Fig. 12c) with the same width throughout, anterior apex not projecting ventrally. Antennal grooves distinct, with lateral margin curved.

*Pterothorax.* Scutellar shield 1.2× as long as wide, sparsely punctured. Elytra widest at anterior fifth, 1.0–1.1× longer than wide, 2.6–2.8× as long as pronotum, 1.2–1.3× as wide as pronotum, surface glabrous, with 10 series of punctures; series 6, 8 and 9 not reaching anterior margin, surface glabrous (Fig. 12d), serial punctures getting slightly smaller lateraly; intervals moderately convex; punctation on interval 1 and odd intervals composed of crescent-shaped setiferous punctures, close to striae denser and intermixed with smaller, transverse non-setiferous punctures; even intervals with non-setiferous punctures only; all interstices without microsculpture. Humeral bulge indistinct. Mesoventral plate (Fig. 12f) narrowly elongate, ca. 5.8× as long as wide, widest at midlength, symmetrically narrowing to both apices, anterior apex pointed, posterior apex rounded, posterior tip slightly overlapping over anterior portion of metaventrite; surface with few sparse punctures. Metaventrite (Fig. 12g) with raised pentagonal area ca. as wide as long, weakly, sparsely, uniformly punctated, without visible setae, bare part not reaching anterior margin of metaventrite; femoral lines absent; lateral parts of metaventrite densely covered by short pubescence.

*Legs.* Femora with sparse shallow punctures ventrally, interstices with weak microsculpture consisting of longitudinal lines; tibial grooves distinct. Tibiae with rather small lateral spines. Metatibiae moderately broad and long, straight, 0.33× as long as elytra, 5× as long as wide. Metatarsus long, 0.86–0.89× as long as metatibia, with just a few short rather stout setae ventrally.

*Abdomen* with five ventrites, first abdominal ventrite (Fig. 12h) about as long as the second and third ventrites together, with distinct median longitudinal carina narrowing posteriad, not projecting posteriorly in both sexes; fifth ventrite with acuminate apex and weakly bulged in both sexes (Fig. 12i).

*Male genitalia.* Median projection of sternite 9 (Fig. 4i) rounded apically, with a pair of subapical setae, base symmetrical. Phallobase (Fig. 4f) almost 1.4× longer than parameres, narrow, parallel sided, base widely rounded, manubrium indistinct. Parameres nearly of the same width in basal 3/4, divergent near apex, rounded and weakly narrowing apically. Median lobe (Fig. 4g) narrow and subparallel throughout, pointed at apex (Fig. 4h), gonopore moderately large, basal portion of median lobe with dorsal plate narrow and simply bifid basally.

###### Variability.

The general dorsal coloration of the pronotum and elytra varies from yellow to dark reddish-brown. In dark specimens, lateral pronotal spots join the large central spot, and the whole anterior part of elytra and the elytral suture are distinctly darkened, with pale areas maintained in humeral area and at sides of scutellar shield. In pale specimens, the lateral pronotal spots are rather small and sometimes very vague and indistinct, and the elytra are completely yellow except base, sutural interval and the posthumeral dark spots.

###### Distribution.

*Cercyon insularis* seems to be widely distributed across Greater and Lesser Antilles, here we are recording it from Cuba, Dominican Republic, Puerto Rico, Grenada, Saint Lucia and Dominica. It seems that all records of *C. variegatus* from the Caribbean (Jamaica, Puerto Rico: Smetana 1978; Dominica: Peck 2006) actually concern *C. insularis*, as we failed to find the true *C. variegatus* in the material examined. For that reason we consider *C. insularis* to occur in Jamaica, although we did not examine any specimens from Jamaica ourselves.

###### Bionomics.

Most of the specimens were collected in cow and horse dung on pastures, in coffee plantations and in tropical forests; few were collected using flight intercept traps.

###### Discussion.

Chevrolat (1863) described *C. insularis* based on a single specimen from Cuba collected by D. F. Poëy and deposited in Chevrolat's collection. On our request to loan this specimen, we received a single specimen standing under the name *C. insularis* in the Chevrolat collection, corresponding well with the original description and marked as a type. In contrast to the data mentioned by Chevrolat (1863), the specimen also bears a label indicating Habana as the place of its origin. Since there is no reason to doubt the type identity of this specimen, we correct the type locality of *C. insularis* to Habana, in agreement with the label data of the holotype.

*Cercyon insularis* was only briefly mentioned once by Gundlach (1891) and its type was not reexamined, therefore its identity remained unclear. Our inspection of the type revealed it corresponds by coloration with what was recorded from the Caribbean as *Cercyon variegatus* Sharp, 1882, by Smetana (1978), Hansen (1999) and Peck (2006). In order to determine the identity of the species present in the Caribbean we studied the lectotype of *C. variegatus* (Fig. 4j–o, deposited in BMNH) and compared it to the type of *C. insularis* and additional recently collected material from Cuba. Based on this comparison, it became clear that Cuban specimens are not conspecific with *C. variegatus*, but belong to a different species indistinguishable from it by external morphology: all dissected Cuban specimens were conspecific, differed from *C. variegatus* by genital morphology, and no other species of the same external coloration was found. We hence consider the Cuban specimens conspecific with the type specimen of *C. insularis*, even though it cannot be dissected because of its poor condition. Both median lobe (Fig. 4g) as well as phallobase and parameres (Fig. 4f) are narrower in *C. insularis* than in *C. variegatus*. The apices of parameres are rounded in *C. insularis*, while they are more acuminate in *C. variegatus* (Fig. 4l). Moreover, the apex of the median lobe of *C. variegatus* has a small flank on each side (Fig. 4n) (Full set of pictures of the lectotype of *C. variegatus* in www.flickr.com/photos/142655814@N07/albums/72157676248390724).

*Cercyon insularis* and *C. variegatus* belong to a species complex corresponding to the *C. variegatus* group of Smetana (1978), distributed from the southern USA to Argentina; the species within this complex can be only distinguished by the morphology of the male genitalia (Arriaga-Varela and Fikáček, pers. observation). Only two species of this species complex have been formally described (*C. variegatus* and *C. insularis*) and the group requires a detailed revision. The species recorded from Suriname as “*Cercyon rishwani*” by Makhan (2004) also belongs to this species complex based on color pattern of the pronotum and the general shape of the aedeagus, but a more detailed comparison with *C. insularis* and *C. variegatus* is impossible based on the description and illustrations provided. “*Cercyon rishwani* Makhan, 2004” is moreover considered a *nomen nudum* (see Short and Hebauer, 2006 for details).

**Figure 8.**
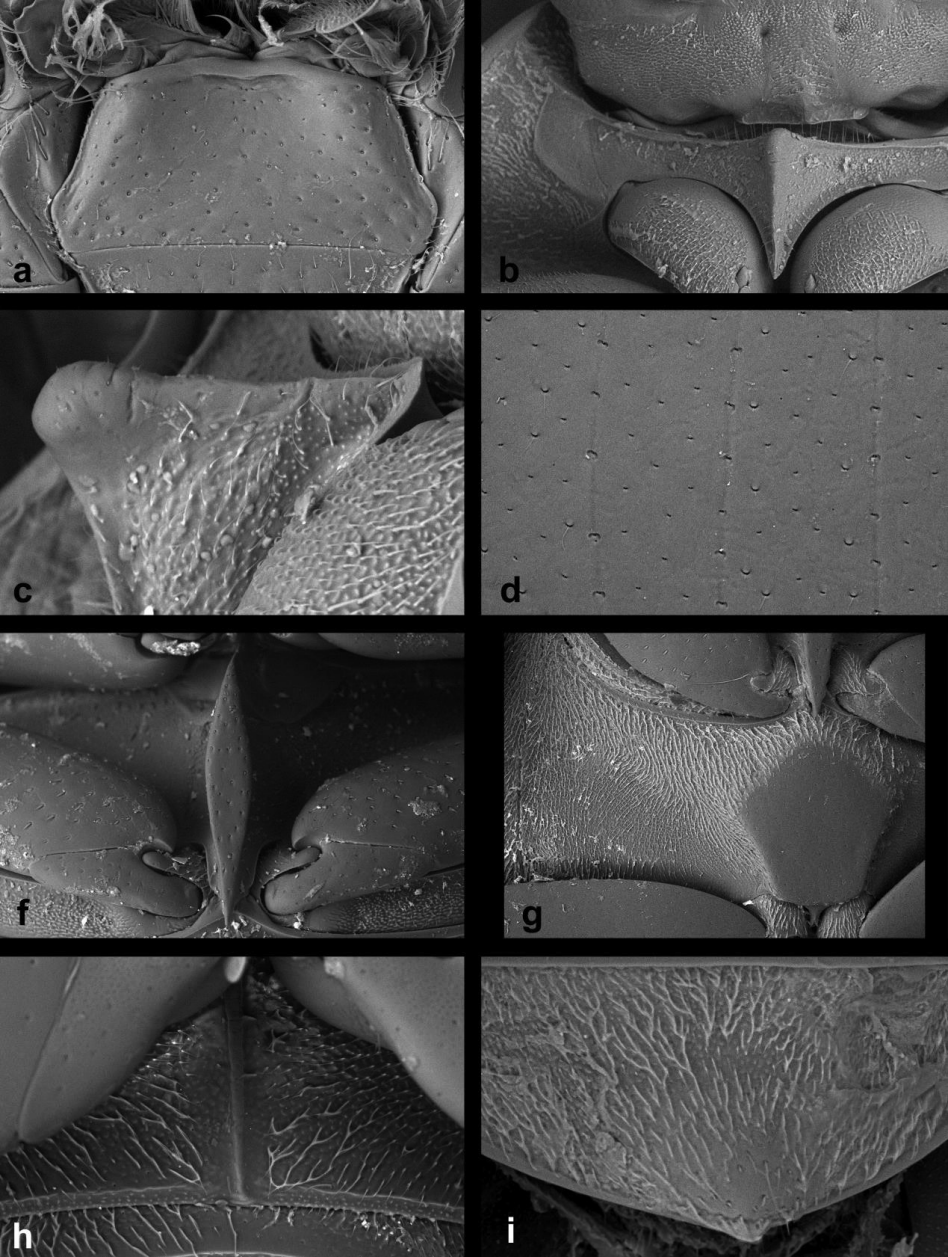
*Cercyon gimmeli* sp. n. **a** mentum **b** prosternum **c** lateral view of median ridge of prosternum **d** detail of elytral surface **f** mesoventral plate **g** metaventrite **h** median ridge of first abdominal ventrite **i** fifth abdominal ventrite.

**Figure 9.**
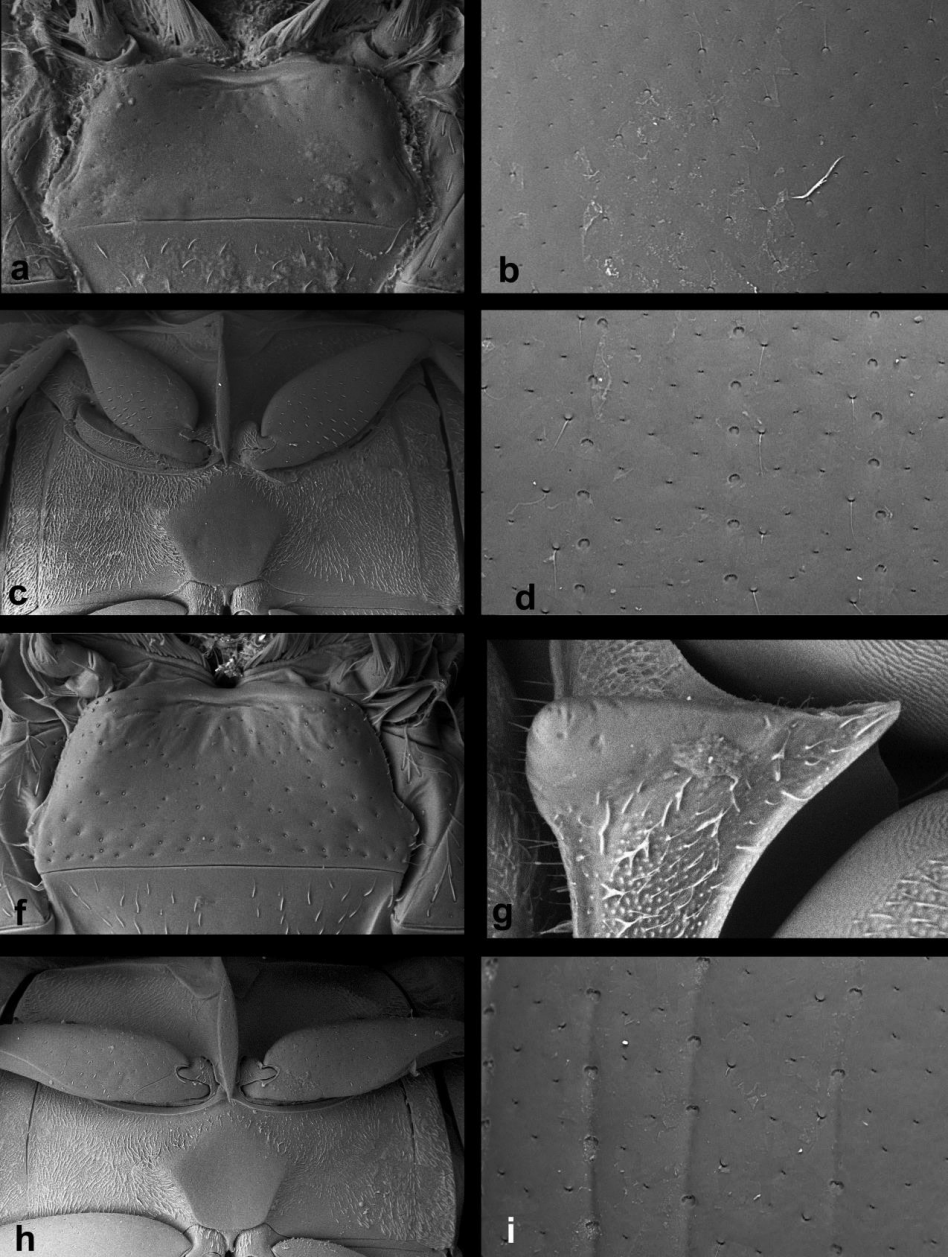
*Cercyon* spp. n. **a–d** *Cercyon armatipenis* sp. n. **f–i** *Cercyon taino* sp. n. **a, f** mentum **b** detail of pronotal surface **c, h** ventral view of pterothorax **d, i** detail of elytral surface **g** lateral view of median ridge of prosternum.

**Figure 10.**
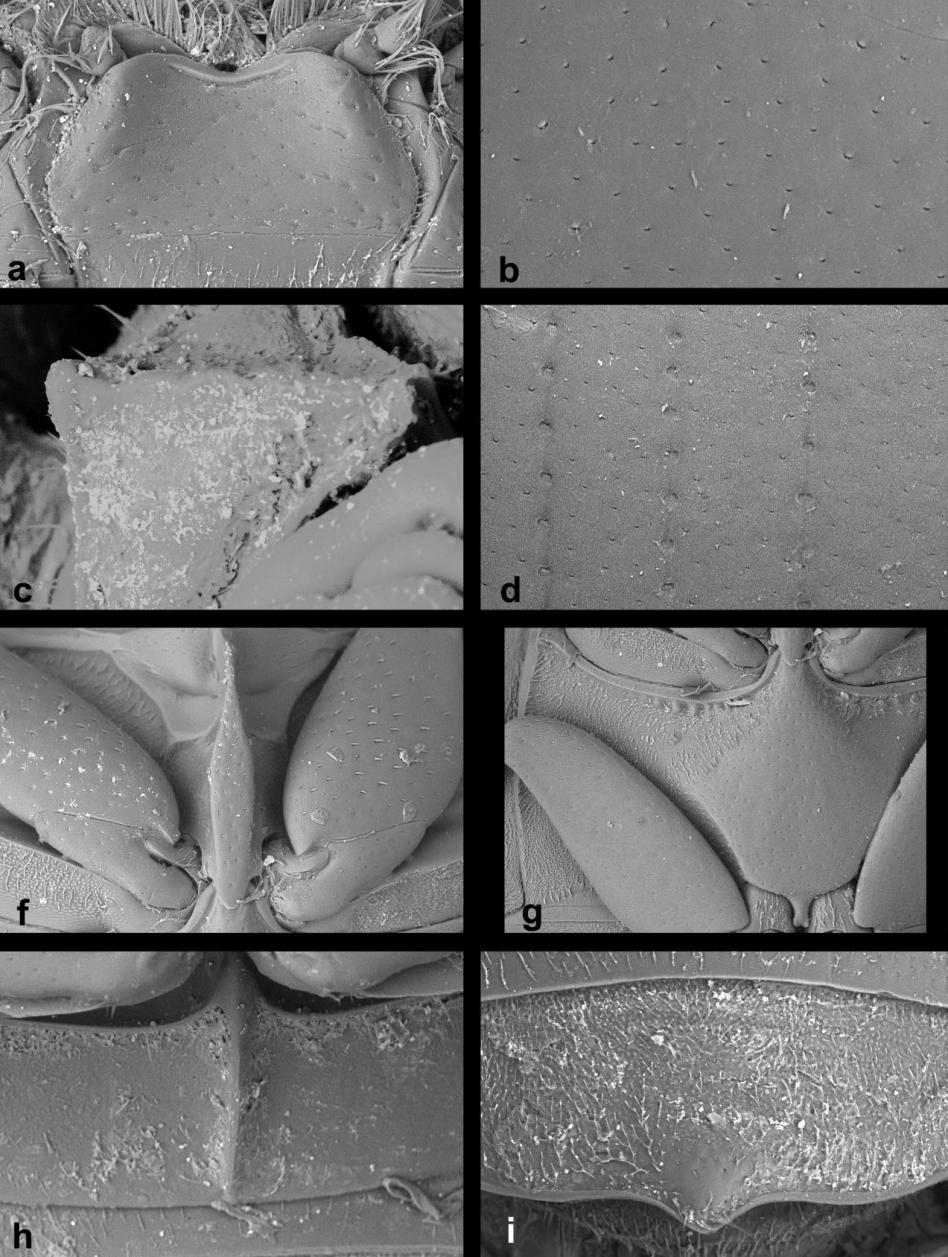
*Cercyon sklodowskae* sp. n. **a** mentum **b** detail of pronotal surface **c** lateral view of median ridge of prosternum **d** detail of elytral surface **f** mesoventral plate **g** metaventrite **h** median ridge of first abdominal ventrite **i** female fifth abdominal ventrite.

**Figure 11.**
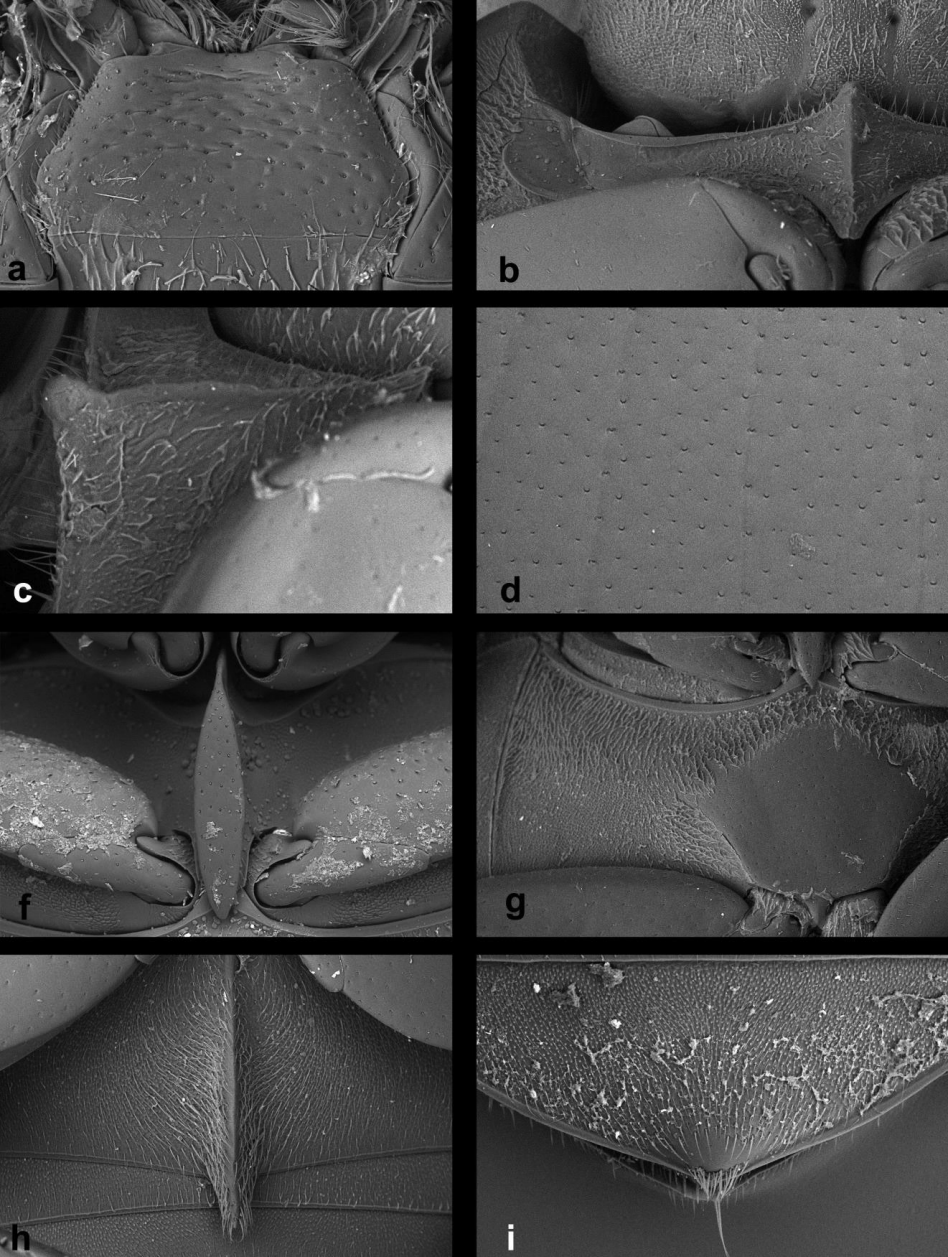
*Cercyon spiniventris* sp. n. **a** mentum **b** prosternum **c** lateral view of median ridge of prosternum **d** detail of elytral surface **f** mesoventral plate **g** metaventrite **h** median ridge of female first abdominal ventrite **i** fifth abdominal ventrite.

**Figure 12.**
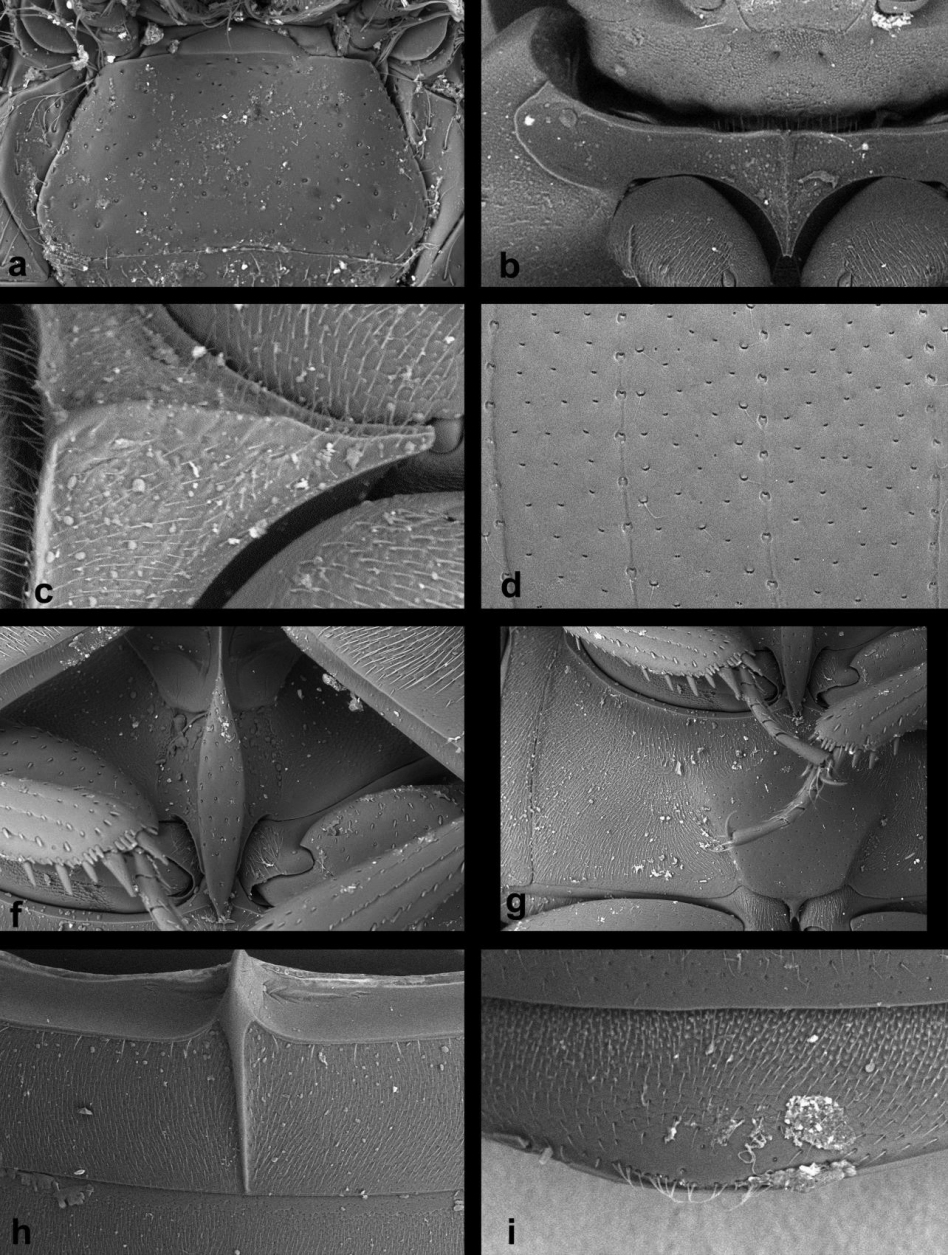
*Cercyon insularis* Chevrolat **a** mentum **b** prosternum **c** lateral view of median ridge of prosternum **d** detail of elytral surface **f** mesoventral plate **g** metaventrite **h** median ridge of female first abdominal ventrite **i** fifth abdominal ventrite.

**Figure 13.**
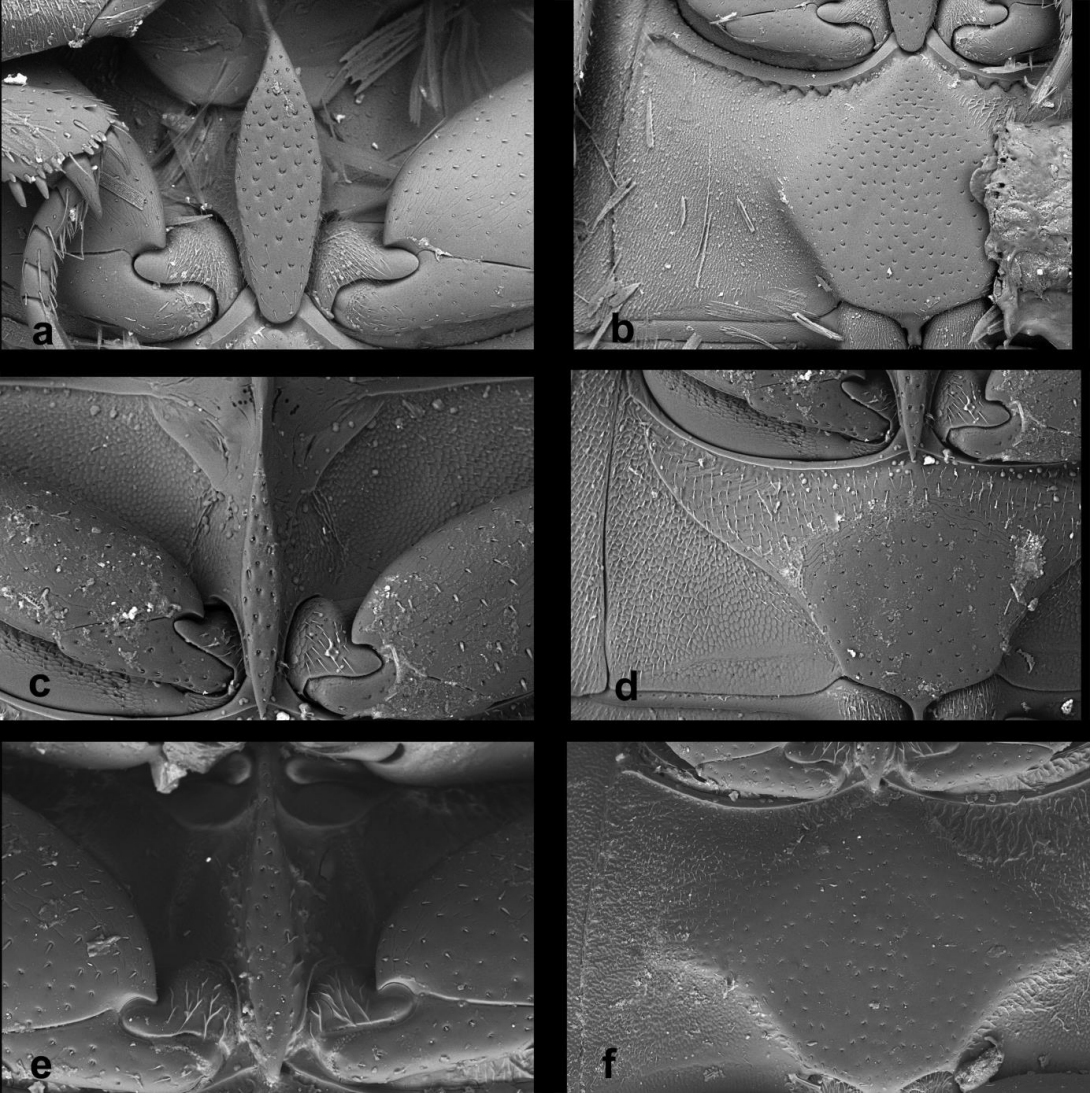
*Cercyon* spp. **a–b** *Cercyon praetextatus* Say **c–d** *Cercyon nigriceps* Marsham **e–f** *Cercyon quisquilius* Linnaeus **a, c, e** mesoventral plate **b, d, f** metaventrite.

#### Larval morphology

##### Cercyon taino sp. n.

Taxon classificationAnimaliaColeopteraHydrophilidae

Figure 14a–f

###### Material examined.

**DOMINICAN REPUBLIC: La Vega**: PN Valle Nuevo, Salto Aguas Blancas, sifting of moist leaf litter in small remnants of montane forest in a small ravine with a spring and on slopes just above the small river, 18°50.60'N, 70°40.68'W, 1655 m a.s.l., 25.viii.2014, leg. Deler, Fikáček & Gimmel (DR21) (2 larvae associated with adults: NMPC; DNA extraction of one larva: MF1261.L).

###### Larval diagnosis.

**Figure 14.**
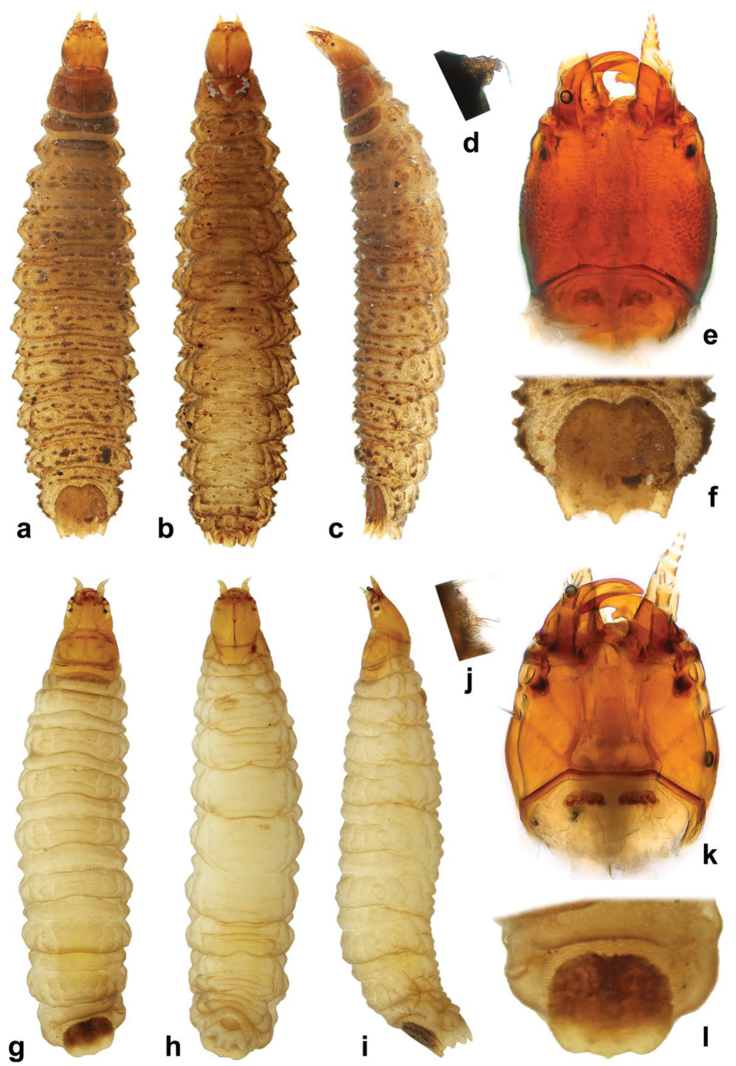
*Cercyon* spp. larval morphology **e–f** *Cercyon taino* sp. n. **g–l** *Cercyon insularis* Chevrolat **a, g** dorsal habitus **b, h** ventral habitus **c, i** lateral habitus **d, j** front leg **e, k** dorsal view of head **f, l** tergite on 8th abdominal segment.

Head capsule (Fig. 14e) longer than wide; cuticle with polygonal microsculpture; head capsule with two “lenses” (anteriorly and posterior of the eye spot) on each side, lateral part of head capsule without apparent group of setae ca. at midlength; clypeolabrum uniformly arcuate at the right side from the setiferous emargination. Metanotum (Fig. 14a) with wide and strongly sclerotized transverse tergite. Legs (Fig. 14d) reduced into two-segmented vestiges. Membranous parts of thorax and abdomen (Fig. 14a–c) covered by long blackish cuticular projections. Abdominal segments acutely lobate laterally, abdominal segments 1-7 each with three transverse rows of low tubercles. Tergite on 8th abdominal segment (Fig. 14f) ca. as long as wide, deeply sinuate on anterior margin, with three slightly acute lobes on posterior margin.

##### Cercyon insularis

Taxon classificationAnimaliaColeopteraHydrophilidae

Chevrolat

Figure 14g–l

###### Material examined.

**PUERTO RICO: Naguabo**: El Yunque National Forest, (southern part), 3.45 km N of Río Blanco at road PR191, in horse excrements on exposed small pasture on the slope of El Yunque massive, 18°14.8'N, 65°47.9'W, 170 m a.s.l., 24.vi.2016, leg. Deler-Hernández, Fikáček & Seidel (PR2a) (2 larvae associated with adults: NMPC; DNA extraction of one larva: MF1731.L)

###### Larval diagnosis.

Head capsule (Fig. 14k) ca. as long as wide; cuticle smooth, without distinct miscrosculpture; head capsule with two “lenses” (anteriorly and posterior of the eye spot) on each side, lateral part of head capsule with apparent group of setae (PA12-14 *sensu* Fikáček et al. 2008) ca. at midlength; clypeolabrum angulate at the right side from the setiferous emargination. Metanotum (Fig. 14g) with very narrow and weakly sclerotized transverse tergite. Legs (Fig. 14g) reduced into one-segmented setiferous tubercle. Membranous parts of thorax and abdomen covered by extremely short and dense whitish microtrichia. Abdominal segments (Fig. 14g–i) without lateral lobes, smooth (i.e. without transverse rows of tubercles) dorsally. Tergite on 8th abdominal segment (Fig. 14l) wider than long, shallowly sinuate on anterior margin, weakly sinuate on posterior margin.

**Figure 15.**
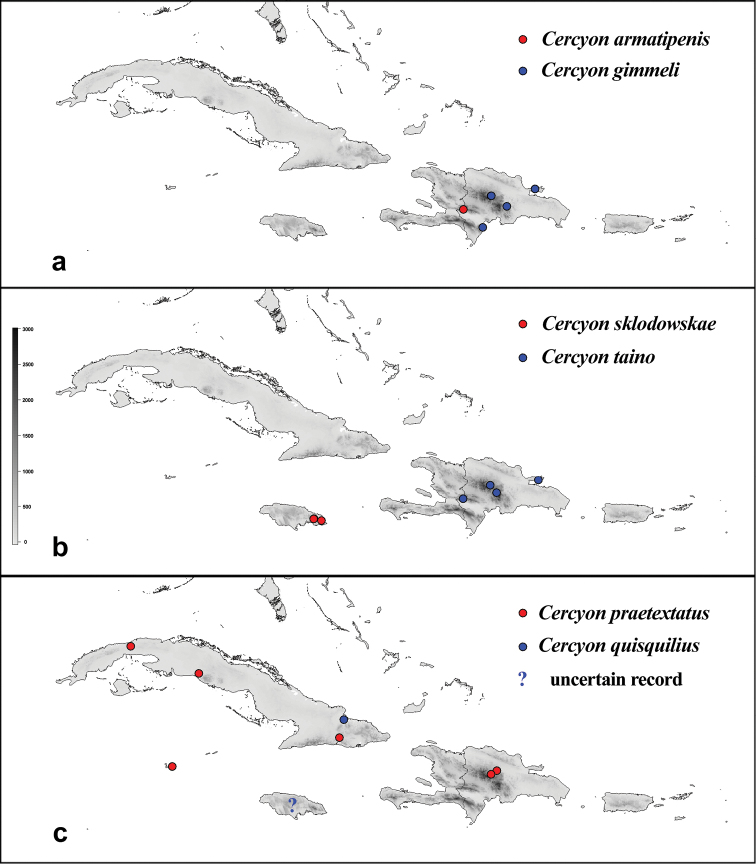
*Cercyon* spp. distribution maps. **a** *Cercyon armatipenis* sp. n. (🔴) *Cercyon gimmeli* sp. n. (🔵) **b** *Cercyon sklodowskae* sp. n. (🔴) *Cercyon taino* sp. n. (🔵) **c** *Cercyon praetextatus* Say (🔴) *Cercyon quisquilius* Linnaeus (🔵).

**Figure 16.**
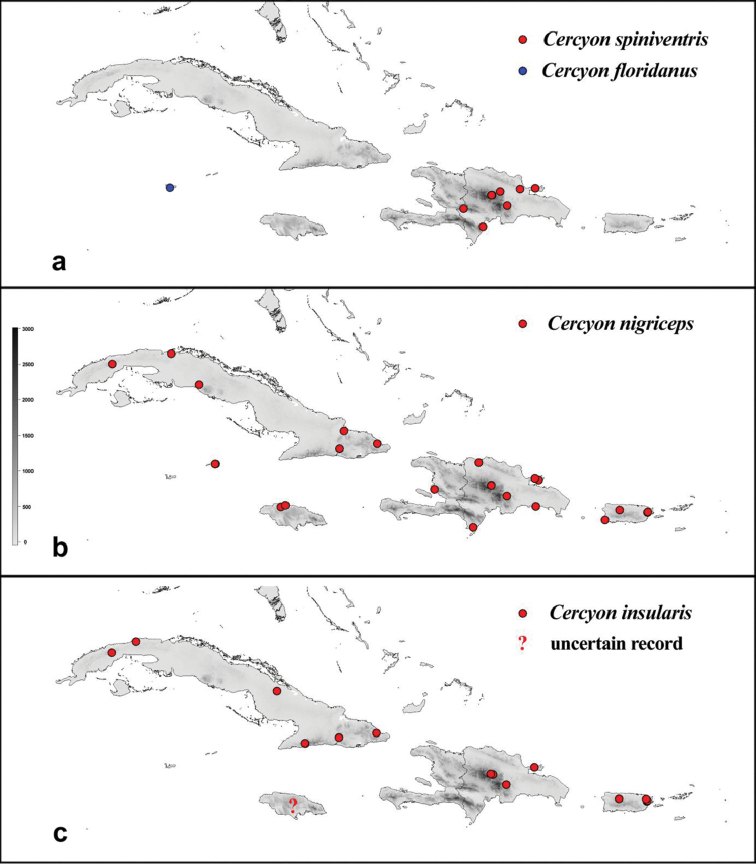
*Cercyon* spp. distribution map. **a** *Cercyon spiniventris* sp. n. (🔴) *Cercyon floridanus* Horn (🔵) **b** *Cercyon nigriceps* Marsham (🔴) **c** *Cercyon praetextatus* Say (🔴) *Cercyon insularis* Chevrolat (🔴).

**Figure 17.**
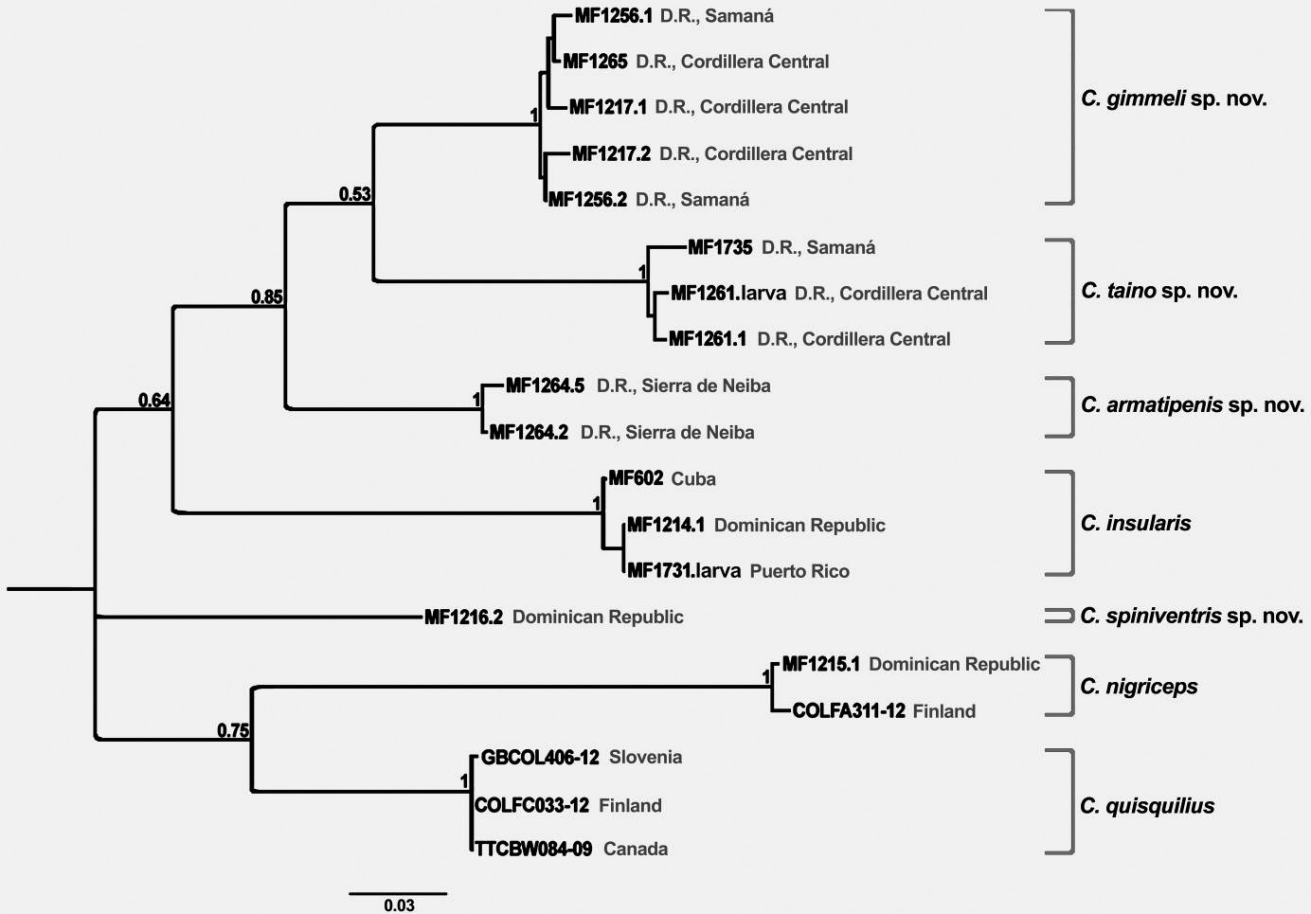
Maximum likelihood tree 1000 bootstrap replicates resulting from the phylogenetic analysis of DNA barcode of 19 specimens of *Cercyon* spp. Newly sequenced Caribbean specimens have the MF codes, other sequences are adopted from BOLD database.

### Analysis of molecular data

Partial COI sequences of the 19 *Cercyon* specimens sampled resulted in a 610 bp alignment. JModelTest (Darriba et al. 2012) determined the GTR+I+G model as best nucleotide substitution model. The resulting maximum likelihood tree revealed 7 clades corresponding to the species as defined by morphological characters (Fig. 17). The statistical support for all species clades containing multiple specimens was high (bootstrap 100%), whereas the backbone of the phylogeny was poorly supported (bootstrap 53–85%), corresponding to the fact that we used a single marker with high substitution rate with limited information content for interspecific phylogeny, especially when introduced (and hence likely not closely related) species were included in the analysis. However, we recovered the *C. gimmeli* species complex as a well-supported monophyletic group, containing three well separated lineages (corresponding to the species as delimited by genital morphology) with mean interspecific distances 9–10 %. Sequenced females of the *C. gimmeli* species complex, morphologically not identifiable to species, were all unambiguously associated with male specimens and included as paratypes. *Cercyon insularis*, *C. spiniventris* and the introduced species were also revealed as well separated lineages in our analysis. The intraspecific genetic distances were low in all species for which multiple specimens were included, ranging between 0.0 to 1.4 %. Larval specimens were unambiguously assigned to co-occuring adults in both cases (larva MF1261.L to *C. taino*, larva MF1731.L to *C. insularis*). The only sequenced specimen of *C. nigriceps* from Puerto Rico is genetically very similar to that of Finland (genetic distance 0.6 %) (See supplementary material 2).

## Discussion

**Faunal composition of *Cercyon* in the Greater Antilles.** Our revision raises the number of *Cercyon* species from six to 10, and shows that the composition of the fauna largely differs from the original ideas: (1) five species, i.e. half of the fauna, are probably single-island endemics (four species in Hispaniola, one in Jamaica) and one additional species (*C. insularis*) seems to be a widespread Caribbean endemic not occurring in the mainland Americas; (2) only two species occurring in the Greater Antilles (*C. praetextatus* and *C. floridanus*) are native to the American continent, of which *C. floridanus* is moreover limited only to Cayman islands and does not occur in the four main islands of Greater Antilles; (3) two introduced Old World species (*C. nigriceps* and *C. quisquilius*) occur in the Greater Antilles, both of which are nowadays also widespread in the American continent (Smetana 1978; Fikáček 2009). The widespread continental *Cercyon variegatus* which was originally recorded from Jamaica and Puerto Rico by Smetana (1978) does not occur in the Caribbean Region at all, and is replaced there by morphologically very similar and likely closely related *C. insularis*.

The comparison of the original ideas about the *Cercyon* fauna and its real composition corresponds to the situation found in many other groups of minute arthropods, which were recently studied in the Greater Antilles. These studies frequently discover higher numbers of single-island endemics than expected, and reveal that some widespread continental species recorded from the Caribbean are in fact endemic species closely related but not identical to the continental ones (e.g., Dziki et al. 2015; Agnarsson et al. 2016).

The probable single-island endemics discovered during this study are distributed in Hispaniola (four species) and Jamaica (one species) only. No new species were discovered in Cuba and Puerto Rico, despite our collecting effort in both islands. Our field work in Puerto Rico was rather short and did not cover all mountain regions. Therefore, we cannot exclude additional discoveries in the island. On the other hand, our sampling effort was highest in Cuba, with intensive field work performed in 2010–2016. Hence we consider the discovery of a new endemic species in Cuba less likely. This puts in contrast Cuba, i.e. the largest Greater Antillean island, with no endemic species, with the smaller Hispaniola, hosting at least four single-island endemics. Moreover, the discovery of *C. armatipenis* sp. n. in a single locality in Sierra de Neiba at the Dominican-Haitian border indicates that additional species may be expected in this species complex in the western part of the island (i.e. Haiti) which was not sampled so far. In the same manner, there is a chance that the only species so far known exclusively from the Lesser Antilles, *Cercyon cribratus* Castelnau (1840), described from Guadeloupe island, could be present in the Greater Antilles. However, we were not able to find any specimen fitting its description in the examined material.

Both the extremely similar morphology and results of the analysis of the COI sequences imply that the *C. gimmeli* species complex forms a monophyletic clade endemic to Hispaniola. The fact that all species of the complex occur sympatrically and no clear geographic pattern can be observed in their ranges suggests that the radiation of this group in Hispaniola may be a result of subdivision of Hispaniola in smaller paleoislands during the Oligocene to Middle Miocene followed by range expansion when the paleoislands got interconnected more recently (Iturralde-Vinent and MacPhee 1999; Iturralde-Vinent 2006; Matos-Maravi et al. 2014). The group would be hence a good model for a further more detailed biogeographic study.

**Novel morphological characters.** Morphological studies of the Greater-Antillean *Cercyon* revealed some characters of adults relevant to species discrimination and identification, but not used before: the presence/absence and shape of the projection of the anterior part of the prosternal medial ridge, and the sexually dimorphic characters of abdominal ventrites found in *C. spiniventris* and *C. sklodowskae*. Females of *C. spiniventris* are characterized by a spine-like process on the first abdominal ventrite (absent in males), which has not been recorded in any other *Cercyon* species so far, but is present in females of the Australian megasternine genus *Cercyodes* Broun (Hansen 1990). Females of *C. sklodowskae* are characterized by modified shape of the fifth abdominal ventrite (compared to simple one in males). We have observed the variation of the shape of the fifth ventrite also in some other *Cercyon* species (without checking their sex). Hence the sexual dimophism in this character may be more widespread than expected.

Interesting discoveries were also made by a brief examination of the larvae of *C. insularis* and *C. taino*. Both larvae are surprisingly quite different from each other, differing especially in the head proportions, extent of leg reduction, shape of the tergite of 8^th^ abdominal segment, and surface vestiture membranous parts of thorax and abdomen. In all these characters, *C. insularis* is more similar to other *Cercyon* larvae described in the literature (*C. quisquilius* and *C. praetextatus*: Archangelsky 2016; *C. melanocephalus* and *C. haemorrhoidalis*: De Marzo 2000; *C. unipunctatus*, *C. pygmaeus* and *C. lateralis*: Schulte 1985). When examining the larval head of both *C. insularis* and *C. taino*, we found two circular areas of thickened transparent cuticle, one situated just in front of the ocular spot and another one just behind it (the areas are also visible as paler spots in lateral view of the head). We suppose that these areas of thickened cuticle may function as lenses. This character was never studied in sphaeridiine larvae and the only information about the ocular region available in the literature concerns the presence and shape of the pigmented “ocular spots”: in larval Megasternini one spot is present, interpreted as an aggregation of all six stemmata or three anterior stemmata only (Hansen and Richardson 1998; Archangelsky 1999). Additional studies are needed to understand the function of this structure and its distribution in the Megasternini and Sphaeridiinae. One lens is present on each side of the head capsule of the larvae of *C. praetextatus* and the unidentified Japanese larvae of *Cercyon* (M. Archangelsky and Y. Minoshima, pers. comm. 2016), and two lenses (one before and one behind the pigmented spot) are present on each side of the head of larval *Sphaeridium*, i.e. the sister-group to the tribe Megasternini (M. Archangelsky, pers. comm.).

The novel characters mentioned above of both adults and larvae are useful for diagnostic purposes, but can also have phylogenetic signal which will help to corroborate the results of the ongoing phylogenetic study of the tribe Megasternini. The differences found between the larvae of *C. insularis* and *C. taino* and the differences in the number of lenses between different megasternine taxa show that larval morphology of the Megasternini is not that uniform as previously believed, and is in need of more studies.

**Subgeneric assignment of the Greater Antilles *Cercyon* species.** All species treated in this paper fall into the concept of *Cercyon* sensu stricto. However, we refrain from assigning them to any subgenus since the systematics of the genus *Cercyon* and allies is currently under study, and previous studies have shown that *Cercyon* in the current concept may be a polyphyletic assemblage of species (e.g. Short and Fikáček 2013). Newly discovered characters of adults and larvae discussed above also indicate that *Cercyon* is much more morphologically heterogeneous than expected, which corresponds to its supposed polyphyly.

## Author contribution

ADH, MF and MS performed the field work; EAV and MF accumulated additional museum material; EAV performed the majority of morphological studies, prepared the first draft and photodocumentation; EAV and MS did the molecular work, analysis of the data and prepared the data for submissions to BOLD; VS, MF and EAV prepared the datasets, wrote and tested the scripts, and submitted the data to BOLD, GBIF and Flickr; all authors commented drafts of the paper at different stages and helped with completing the manuscript for submission.

## Supplementary Material

XML Treatment for Cercyon

XML Treatment for Cercyon gimmeli

XML Treatment for Cercyon armatipenis

XML Treatment for Cercyon taino

XML Treatment for Cercyon sklodowskae

XML Treatment for Cercyon floridanus

XML Treatment for Cercyon praetextatus

XML Treatment for Cercyon spiniventris

XML Treatment for Cercyon nigriceps

XML Treatment for Cercyon quisquilius

XML Treatment for Cercyon insularis

XML Treatment for Cercyon taino

XML Treatment for Cercyon insularis

## Supplementary materials

Supplementary material 1 Darwin Core formatted distribution data Data type: species distribution Explanation note: The Excel file includes all fields (columns) defined by the DarwinCore (DwC) format relevant to our data (headers highlighted in green) and ordered in a customized way (order of columns is not important, columns are identified by their header names that need to remain unchanged). We added a few fields (columns), which are not present in DwC, to include additional information needed in our case (about specimens in a DNA-tissue collection, DNA extracts done, and DNA barcodes), or to make the data input more convenient (two separate columns for starting and ending event dates, from which the date range in DwC format is formed and saved in the eventDate field); headers of all additional fields are marked in grey in Suppl. material 1. This dataset is made available under the Open Database License (http://opendatacommons.org/licenses/odbl/1.0/). The Open Database License (ODbL) is a license agreement intended to allow users to freely share, modify, and use this Dataset while maintaining this same freedom for others, provided that the original source and author(s) are credited.Emmanuel Arriaga-Varela, Matthias Seidel, Albert Deler-Hernández, Viktor Senderov, Martin Fikáček

Supplementary material 2 Genetic distances within and between species of Caribbean *Cercyon* Data type: molecular data Explanation note: Mean genetic distances based on barcode CO1 sequences within (in grey fields) and between species (in white fields) calculated using the Maximum Composite Likelihood model as implemented in MEGA7. Standard error estimates (in square brackets) obtained by bootstraping (1000 replicates).@. This dataset is made available under the Open Database License (http://opendatacommons.org/licenses/odbl/1.0/). The Open Database License (ODbL) is a license agreement intended to allow users to freely share, modify, and use this Dataset while maintaining this same freedom for others, provided that the original source and author(s) are credited.Emmanuel Arriaga-Varela, Matthias Seidel, Albert Deler-Hernández, Viktor Senderov, Martin Fikáček

Supplementary material 3 DwC2BOLD script Data type: script Explanation note: We used this script to automatically transform the DwC-formatted specimen data into the format required for the submission of specimen data to BOLD. The script uses the Barcode field (column) in our DwC spreadsheet to filter out the specimens that were barcoded and that need to be submitted to BOLD (notice that the Barcode field was added by us and is not a part of the DwC format!). Due to the slightly different data format used in DwC and BOLD, it is not possible to convert all information automatically, and the resulting BOLD-formatted data should be checked before submission. The script displays alerts for fields in which a mismatch between DwC and BOLD is possible. The script is available in Suppl. material 3 as a zip archive containing an R script, a PDF rendering and an HTML rendering. This dataset is made available under the Open Database License (http://opendatacommons.org/licenses/odbl/1.0/). The Open Database License (ODbL) is a license agreement intended to allow users to freely share, modify, and use this Dataset while maintaining this same freedom for others, provided that the original source and author(s) are credited.Emmanuel Arriaga-Varela, Matthias Seidel, Albert Deler-Hernández, Viktor Senderov, Martin Fikáček

Supplementary material 4 DwC2Map script Data type: script Explanation note: The script reads the decimal GPS data from our DwC Excel file and creates a dataframe with all observations for each species. In the next step it allows to construct the maps of the area from freely available on-line data and map the occurrences of thes species on these maps. The maps in Figs 15–16 were generated using this script. The script is available in Suppl. material 4 as a zip archive containing an R script, a PDF rendering and an HTML rendering. This dataset is made available under the Open Database License (http://opendatacommons.org/licenses/odbl/1.0/). The Open Database License (ODbL) is a license agreement intended to allow users to freely share, modify, and use this Dataset while maintaining this same freedom for others, provided that the original source and author(s) are credited.Emmanuel Arriaga-Varela, Matthias Seidel, Albert Deler-Hernández, Viktor Senderov, Martin Fikáček
