# Supplementary material for: A review of the Cercyon Leach (Coleoptera, Hydrophilidae, Sphaeridiinae) of the Greater Antilles
Source: Zookeys. 2017 Jun 21;(681):39–93. doi: 10.3897/zookeys.681.12522 (PMC5523880; doi:10.3897/zookeys.681.12522)
Supplement: Supplementary material 3 — DwC2BOLD script [file zookeys-681-039-s003.html]

Conversion of DarwinCore formatted tabular data into BOLD specimen template format


# Conversion of DarwinCore formatted tabular data into BOLD specimen template format

#### *Viktor Senderov, Emmanuel Varriaga Varela, Martin Fikacek*

#### *2017-02-21*

# Introduction

This is an R Notebook documenting the conversion of DarwinCore formatted tabular data into the BOLD specimen template format.

DarwinCore formatted tabular data should be an Excel sheet or a CSV file, where the column headings correspond to the DarwinCore terms. After the execution of the code here, the user gets tabular data (Excel or CSV) formatted according to the BOLD specimen template.

Please change adapt this code as necessary for your needs before running it!

This script requires the `xlsx` library!

```
library(xlsx)
```

# Input

## CSV Case

If you want the input to be DwC-formatted CSV file, please set the variable `csv_DwC` to the proper filename. Note that for Windows, you separate directories with double backslash, `\\`. Please, export it as CSV by using the following delimeters:

- Field delimeter: `,`
- Text delimeter: `"`

Don’t save any formulas, instead save the calculated values.

```
csv_DwC  = "/home/viktor/Work/Fikacek_paper/Viktor/dwc.csv"
DwC = read.csv(csv_DwC, header = TRUE, stringsAsFactors = FALSE,
                                                            encoding = "UTF-8")
```

## Excel case

If you want the input to be a DwC Excel file set `excel_file_DwC`:

```
excel_file_DwC  = "/home/viktor/Work/Fikacek_paper/Viktor/"
DwC = read.xlsx(excel_file_DwC, "Occurrence", header = TRUE,
                                                     stringsAsFactors = FALSE )
```

# Functions

The following are necessary processing functions in both cases. Please, source them:

```
# Checks for presence of required fields.
check_required_fields = function ( Required_Fields, The_Data ) {
  status = TRUE
  for ( field in Required_Fields ) {
    local_status = FALSE
    for ( f in field ) {
      if ( ( f %in% names( The_Data ) ) ) {
        cat( paste("Found", f, "\n"))
        local_status = TRUE
      }
    }
    if ( !local_status ) {
      cat( "ERROR: One of ")
      cat(field)
      cat(" is required!")
      status = FALSE
    }
  }
  return ( status )
}

# Check for bad matches
check_bad_matches = function ( Bad_matches, The_Data ) {
  status = FALSE
  for ( field in Bad_matches ) {
    for ( f in field ) {
      if ( ( f %in% names( The_Data ) ) ) {
        cat( paste("Warning Found bad match", f, "\n"))
        status = TRUE
      }
    }

  }
  return ( status )
}

# Extends DwC dataframe with missing fields (skipping NO_MATCH)
extend_missing_fields2 = function ( Fields, DwC ) {
  dummy_col = rep(NA, nrow(DwC))
  New_Frame = list()
  for(field in unlist ( Fields ) ) {
    if ( field %in% names( DwC ) ) {
      New_Frame [[ field ]] = DwC[[ field ]]
    }
    else if ( field != "NO_MATCH" ) {
      New_Frame[[ field ]] = dummy_col
    }
  }
  New_Frame = as.data.frame ( New_Frame, stringsAsFactors = FALSE )
  return( New_Frame )
}

# Convert to BOLD
create_bold_dataframe = function ( Fields, DwC ) {
  DwC[is.na(DwC)] = ""
  dummy_col = rep("", nrow(DwC))
  BOLD = list()
  for ( key in names(Fields) ) {
    field = Fields[[key]]
    if ( length(field) == 1 && field == "NO_MATCH") {
      BOLD[[ key ]] = dummy_col
    }
    else {

      current_col = do.call( paste,  DwC[field] )
      BOLD[[ key ]] = current_col
    }
  }
  BOLD = as.data.frame(BOLD, stringsAsFactors = FALSE)
  names(BOLD) = names(Fields)
  return(BOLD)
}

# Processes Types
process_types = function ( a_Dataframe )
{
  vouchered_types = c("holotype", "paratype", "lectotype", "paralectotype", "syntype", "type")
  for ( r in 1:nrow(a_Dataframe) ) {
    if( any( vouchered_types %in% tolower(a_Dataframe[r, "Notes"]) ) ) {
      a_Dataframe [ r, "Voucher Status"] = "Museum Vouchered:Type"
    }
    else {
      a_Dataframe [ r, "Voucher Status"] = ""
      #a_Dataframe [ r, "Notes"] = ""
    }
  }
  return ( a_Dataframe )
}
```

# Mapping

The following is the formal definition of the mapping between DwC and BOLD that we’ve come up with. The mapping can be changed if you find more appropriate bindings. Bear in mind, that when a BOLD field is matched to a vector of DwC fields, this means that the mapping is not one to one and two DwC fields will be somehow concated to form one BOLD field. Currently, the script does simple concatenation but we believe that things like averages, separation by comas, and others can be implemented simply. Furthermore `NO_MATCH` fields will be filled by empty vectors.

```
# This is the mapping between the BOLD fields and the DwC fields
Mapping =
          list(    "Sample ID" = "materialSampleID",
                    "Field ID" = "fieldNumber",
                   "Museum ID" = "catalogNumber",
             "Collection Code" = "collectionCode",
         "Institution Storing" = "institutionCode",
                      "Phylum" = "phylum",
                       "Class" = "class",
                       "Order" = "order",
                      "Family" = "family",
                   "Subfamily" = "NO_MATCH",
                       "Genus" = "genus",
                     "Species" = "specificEpithet",
                  "Identifier" = "identifiedBy",
            "Identifier Email" = "NO_MATCH",
      "Identifier Institution" = "NO_MATCH",
       "Identification Method" = "NO_MATCH",
              "Taxonomy Notes" = "identificationRemarks",
                         "Sex" = "sex",
                "Reproduction" = "reproductiveCondition",
                  "Life Stage" = "lifeStage",
                  "Extra Info" = "occurrenceRemarks",
                       "Notes" = "typeStatus",
              "Voucher Status" = "NO_MATCH",
           "Tissue Descriptor" = "NO_MATCH",
             "Associated Taxa" = "associatedTaxa",
        "Associated Specimens" = c("associatedOccurrences",
                                   "associatedOrganisms"),
               "External URLs" = "associatedReferences",
                  "Collectors" = "recordedBy" ,
             "Collection Date" = "eventDate" ,
             "Country/Ocean"   = c("country", "waterBody"),
              "State/Province" = "stateProvince",
                      "Region" = "county",
                      "Sector" = "NO_MATCH",
                  "Exact Site" = c("locality", "verbatimLocality"),
                    "Latitude" = "decimalLatitude",
                   "Longitude" = "decimalLongitude",
                   "Elevation" = "minimumElevationInMeters",
                       "Depth" = "verbatimDepth",
         "Elevation Precision" = "NO_MATCH",
             "Depth Precision" = "NO_MATCH",
                  "GPS Source" = "georeferenceSources",
         "Coordinate Accuracy" = "coordinatePrecision",
                  "Event Time" = "eventTime",
    "Collection Date Accuracy" = "NO_MATCH",
                     "Habitat" = "habitat",
           "Sampling Protocol" = "samplingProtocol",
            "Collection Notes" = "eventRemarks",
                   "Site Code" = "locationID",
         "Collection Event ID" = "eventID" )

# This the separation of the BOLD fields into subcategories
Fields_BOLD = list( required_fields = c("Sample ID",
                         "Field ID",
                         "Museum ID",
                         "Institution Storing",
                         "Phylum",
                         "Class",
                         "Order",
                         "Family",
                         "Genus",
                         "Species",
                         "Country/Ocean"),

                    bad_matches = c("Reproduction",
                                    "Voucher status",
                                    "External URLs",
                                    "Exact Site",
                                    "Elevation",
                                    "Depth",
                                    "Site Code" ),

                    voucher_info = c("Sample ID",
                                     "Field ID",
                                     "Museum ID",
                                     "Collection Code",
                                     "Institution Storing" ),

                    taxonomy = c( "Sample ID",
                                  "Phylum",
                                  "Class",
                                  "Order",
                                  "Family",
                                  "Subfamily",
                                  "Genus",
                                  "Species",
                                  "Identifier",
                                  "Identifier Email"),

                    specimen_details = c("Sample ID",
                                         "Sex",
                                         "Reproduction",
                                         "Life Stage",
                                         "Extra Info",
                                         "Notes",
                                         "Voucher Status",
                                         "Tissue Descriptor",
                                         "Associated Taxa",
                                         "Associated Specimens",
                                         "External URLs"),

                    collection_data = c("Sample ID",
                                        "Collectors",
                                        "Collection Date",
                                        "Country/Ocean",
                                        "State/Province",
                                        "Region",
                                        "Sector",
                                        "Exact Site",
                                        "Latitude",
                                        "Longitude",
                                        "Elevation",
                                        "Depth",
                                        "Elevation Precision",
                                        "Depth Precision",
                                        "GPS Source",
                                        "Coordinate Accuracy",
                                        "Event Time",
                                        "Collection Date Accuracy",
                                        "Habitat",
                                        "Sampling Protocol",
                                        "Collection Notes",
                                        "Site Code",
                                        "Collection Event ID") )
```

# Processing

First, checm for the required fields:

```
success = check_required_fields( Mapping[Fields_BOLD$required_fields], DwC )
stopifnot( success )
check_bad_matches( Mapping[Fields_BOLD$bad_matches], DwC )
```

Then extend the DwC data frame with the missing fields:

```
DwC = extend_missing_fields2 (Mapping, DwC )
```

Now create the BOLD dataframe:

```
BOLD = process_types( create_bold_dataframe( Mapping, DwC ) )
```

Now create the individual BOLD sheets:

```
Voucher_Info = BOLD[ (BOLD$`Sample ID`) != "", Fields_BOLD$voucher_info]
Taxonomy = BOLD[ (BOLD$`Sample ID`) != "", Fields_BOLD$taxonomy]
Specimen_Details = BOLD[ (BOLD$`Sample ID`) != "", Fields_BOLD$specimen_details]
Collection_Data = BOLD[ (BOLD$`Sample ID`) != "", Fields_BOLD$collection_data]
```

We’re ready to output!

# Output

Here’s where you can download the BOLD Specimen Submission Template from:

(http://www.boldsystems.org/index.php/resources/handbook?chapter=3\_submissions.html) [http://www.boldsystems.org/index.php/resources/handbook?chapter=3\_submissions.html].

## CSV

If you want the output to be a set of BOLD-compatible CSV files, each corresponding to a sheet inside the BOLD Excel workbook, then set `directory_BOLD` to the directory where the files should be output:

```
directory_BOLD = "/home/viktor/Work/Fikacek_paper/Viktor/"
# add the BOLD object to the voucher sheet
write.csv( Voucher_Info, 
           file = paste(directory_BOLD, "/voucher_info.csv", sep = ""),
           row.names = FALSE)
write.csv( Taxonomy,
           file = paste(directory_BOLD, "/taxonomy.csv", sep = ""),
           row.names = FALSE)
write.csv( Specimen_Details,
           file = paste(directory_BOLD, "/specimen_details.csv", sep = ""),
           row.names = FALSE)
write.csv( Collection_Data,
           file = paste(directory_BOLD, "/collection_data.csv", sep = ""),
           row.names = FALSE)
```

## Excel

If you want the output to be a BOLD Excel file, set `excel_file_BOLD`:

```
excel_file_BOLD = "~/home/viktor/Work/Fikacek_paper/Viktor/"
# Load the BOLD workbook and write sheets to it
workbook_BOLD = loadWorkbook( excel_file_BOLD )
sheets_BOLD = getSheets( workbook_BOLD )

# add the BOLD object to the voucher sheet
addDataFrame( Voucher_Info, sheets_BOLD$`Voucher Info`, col.names = FALSE,
                                              row.names = FALSE, startRow = 3)
addDataFrame( Taxonomy, sheets_BOLD$Taxonomy, col.names = FALSE,
                                              row.names = FALSE, startRow = 3)
addDataFrame( Specimen_Details, sheets_BOLD$`Specimen Details`, col.names = FALSE,
                                              row.names = FALSE, startRow = 3)
addDataFrame( Collection_Data, sheets_BOLD$`Collection Data`, col.names = FALSE,
                                              row.names = FALSE, startRow = 3)
saveWorkbook( workbook_BOLD, excel_file_BOLD)
```
