# Supplementary material for: A review of the Cercyon Leach (Coleoptera, Hydrophilidae, Sphaeridiinae) of the Greater Antilles
Source: Zookeys. 2017 Jun 21;(681):39–93. doi: 10.3897/zookeys.681.12522 (PMC5523880; doi:10.3897/zookeys.681.12522)
Supplement: Supplementary material 4 — DwC2Map script [file zookeys-681-039-s004.html]

Construction of z distribution map from a dataset in MS Excel


# Construction of z distribution map from a dataset in MS Excel

#### *Martin Fikacek, Emmanuel Arriaga-Varela, Viktor Senderov*

#### *2017-02-21*

# Introduction

This is an R Notebook with a script used for construction of species distribution maps for the paper by Arriaga-Varela et al. (2017): “A review of the Cercyon (Coleoptera: Hydrophilidae: Sphaeridiinae) of the Greater Antilles.”

The script reads the Excel-based tabular data and constructs the map using open-access on-line data.

There are many other ways how to construct map outputs using freely available data using R, google for alternatives if needed.

Please, change and adapt this code as necessary for your needs before running it!

# 1. Reading the data from the Excel file

Each record in the Excel file should include a species name and the decimal GPS data (longitude and latitude, each in a separate column). We used a [DarwinCore] (http://tdwg.github.io/dwc/terms/index.htm) formatted Excel spreadsheet in our case, but any Excel datasheet contaning the above three columns can be used.

Reading of data from Excel requires the `xlsx` library:

```
options(warn=-1)
library(xlsx)
```

```
## Loading required package: rJava
```

```
## Loading required package: xlsxjars
```

Please, give the full path to the Excel file with your data. Note that for Windows, you separate directories with double backslash, `\\`:

```
excel_file_DwC  =
  "/home/viktor/Work/Fikacek_paper/DwC_CaribbeanCercyon_occurrenceData.xlsx"
```

Please specify the number of the column with the species name, decimal latitude, and decimal longitude here (in this order):

```
cols = c (15, 33, 34)
```

The following chunk reads the selected columns from the Excel file, and creates a dataframe carrying the species name for each species included in tha dataset:

```
DwC = read.xlsx(excel_file_DwC, "Occurrence", header = TRUE,
                stringsAsFactors = FALSE, colIndex = cols)

speciesList <- unique(DwC[1])
SpeciesVector <- sort(as.vector(speciesList[, 1]))
SpeciesNumber <- nrow(speciesList)
for (i in 1:SpeciesNumber)
  {
    species <- SpeciesVector[i]
    speciesSubset <- subset(DwC, DwC[,1] == species)
    SpeciesSubsetUnique <- unique (speciesSubset[c(3,2)])
    assign(paste0(species), SpeciesSubsetUnique)
  }
```

This command, provided column numbers were correct, will have generated a bunch of variables in the global environment corresponding to each species. E.g. “floridanus”, “gimelli”, etc. Also each such variable should have two columns: one for longitude and one for latitude. Make sure you check this!

# 2. Construction of the map with altitudes from GLOBE data

### Input

We used the freely available global elevation data available at the GLOBE webpage. Before starting to construct the map it is needed to download the data and the header files. The world data are divided into several tiles, download all tiles needed for your map.

Download the global elevation data from this site: https://www.ngdc.noaa.gov/mgg/topo/gltiles.html

Add the extension `.bil` to each file (so you will have e.g. `a10g.bil`).

Download the headers for the elevation files here: https://www.ngdc.noaa.gov/mgg/topo/elev/esri/hdr/ and place it to the same folder as the `.bil` file.

This script requires the following libraries. If you don’t find the packages, you can install them via `install.packages()`.

```
library (raster)
```

```
## Loading required package: sp
```

```
library (maptools)
```

```
## Checking rgeos availability: FALSE
##      Note: when rgeos is not available, polygon geometry     computations in maptools depend on gpclib,
##      which has a restricted licence. It is disabled by default;
##      to enable gpclib, type gpclibPermit()
```

```
library (maps)
```

### Loading the altitude data

#### Easy way (try it first)

Set the directory with your `.bil` and `.hdr` files:

```
elevation_dir = "/home/viktor/Data/Global_Elevation_Data"
```

If a single tile covers the area you need to map, load the data from this tile (change the tile name if needed).

Note, we are adding the full path to the elevation file via `paste()`, as `setwd()` is not recommended for R notebooks.

```
map.full <- raster ( paste(elevation_dir, "f10g.bil", sep = "/"))
```

If you need to cover the area divided between multiple tiles, load all needed tiles and combine their data. This can take a while even on a 2016 workstation, mostly CPU load!

```
map.e <- raster ( paste(elevation_dir, "e10g.bil", sep = "/" ))
map.f <- raster ( paste(elevation_dir, "f10g.bil", sep = "/" ))
map.i <- raster ( paste(elevation_dir, "i10g.bil", sep = "/" ))
map.j <- raster ( paste(elevation_dir, "j10g.bil", sep = "/" ))
map.full <- merge(map.e, map.f, map.i, map.j)
```

Now specify the limits of your map as an extent in this order: min longitude, max longitude, min latitude, max latitude:

```
e <- extent (-86, -63, 17, 25)
map.final <- crop (map.full, e)
```

#### If the above way does not work properly

In some systems this command is unable to properly set up the datatype of the raster object, so we need to set it as well. Also, as we don’t want to display negative depths, we need to reclassify them as `NaN`.

Set the directory with your `.bil` and `.hdr` files:

```
elevation_dir = "/home/viktor/Data/Global_Elevation_Data"
```

The case when single tile covers your area:

```
map.full <- raster::raster ( paste(elevation_dir, "f10g.bil", sep = "/"))
dataType(map.full) <- "INT2S"
```

The case when you need to read data from multiple tiles and combine them. This can take a while even on a 2016 workstation, mostly CPU load!

```
map.e <- raster::raster ( paste(elevation_dir, "/e10g.bil", sep = "" ))
map.f <- raster::raster ( paste(elevation_dir, "/f10g.bil", sep = "" ))
map.i <- raster::raster ( paste(elevation_dir, "/i10g.bil", sep = "" ))
map.j <- raster::raster ( paste(elevation_dir, "/j10g.bil", sep = "" ))
dataType(map.e) <- "INT2S"
dataType(map.f) <- "INT2S"
dataType(map.i) <- "INT2S"
dataType(map.j) <- "INT2S"
map.full <- raster::merge(map.e, map.f, map.i, map.j)
```

Now specify the limits of your map as an extent in this order: min longitude, max longitude, min latitude, max latitude.

```
e <- extent (-86, -63, 17, 25)
map.cropped <- crop (map.full, e)
```

To delete the sea, we need to reclassify - this function deletes all altitudes lower than -1 meter from your dataset.

```
map.final <- reclassify( map.cropped, c(-Inf, -1, NaN))
```

### Plotting the map

Now you can plot the map using user-specified color palette - in this case in grey scale:

```
topoColors <- gray(80:5/90)
plot (map.final, col=topoColors)
```

Alternative color shadings may be used, here is an example of color output (specify number of colors and transparency in the parentheses after `terrain.colors`):

```
plot (map.final, col=terrain.colors(15, 0.9))
```

# 3. Adding country boundaries from DIVA-GIS

### Input

Download the shapefiles (.shp) of the borders you want to plot for example from here: http://www.diva-gis.org/Data

You may download complete border dataset or particular countries. Country-based data also offer borders of lower administration units (provinces, states, districts).

Country borders (all in a single file) may be also downloaded from here - it is the `countries_shp.zip` file: https://github.com/gisgraphy/gisgraphy/tree/master/data

### Drawing the borders

Use the script below to load the borders from the .shp files you downloaded and draw the borders on your elevation map created above.

```
country_data = "/home/viktor/Data/Global_Elevation_Data/countries_shp"
borders  <- readShapeSpatial ( paste0( country_data, "/countries.shp") )
plot (map.final, col=terrain.colors(15, 0.5))
plot (borders, add=TRUE, lwd=1)
```

# 4. Plotting the occurrence data

Now you can map the occurrence data loaded from Excel on the map you created. Specify the species name of the species you want to plot.

You can specify the symbols using these parameters:

- `pch` - shape of the symbol
- `cex` - size of the symbol
- `col` - color of the border line
- `lwd` - width of the border line
- `bg` - inside color of the symbol

For possible shapes, see e.g. here: http://www.statmethods.net/advgraphs/parameters.html

We used the following setting for screen view of the distribution data. Here, the `points` command takes as its argument a variable generated in step 1, containing the distribution data for whatever species you want to add to the plot. Feel free to change with your own data!

```
plot (map.final, col=terrain.colors(15, 0.5))
plot (borders, add=TRUE, lwd=1)
points (armatipenis, pch=21, cex=2, col="black", bg="red", lwd=1)
points (gimmeli, pch=21, cex=2, col="black", bg="blue", lwd=1)
```

# 5. Exporting the map in publication quality:

For publication purposes, we first plotted the map with thicker border lines (`lwd=2` in `plot` command) and larger distribution symbols (`cex=4` and `lwd=3` in `points` command).

```
topoColors <- gray(80:5/90)
plot (map.final, col=topoColors)
plot (borders, add=TRUE, lwd=2)
points (armatipenis, pch=21, cex=4, col="black", bg="red", lwd=3)
points (gimmeli, pch=21, cex=4, col="black", bg="blue", lwd=3)
```

Then we exported the resulting map (which looks horrible in the Plot window of R Studio) using the `Export > Save as Image` command, setting 3 times larger size in pixels than the default one and saving the picture in TIFF format. The border lines and symbols will look OK in the resulting TIFF file.
